# Fine-scale movement of northern Gulf of Mexico red snapper and gray triggerfish estimated with three-dimensional acoustic telemetry

**DOI:** 10.1038/s41598-022-18451-x

**Published:** 2022-08-22

**Authors:** Erin C. Bohaboy, Shannon L. Cass-Calay, William F. Patterson

**Affiliations:** 1 Fisheries and Aquatic Sciences, School of Forest, Fisheries, and Geomatics Sciences, University of Florida, 7922 NW 71st Street, Gainesville, FL 32653 USA; 2 National Marine Fisheries Service, Pacific Islands Fisheries Science Center, 1845 Wasp Boulevard, Building 176, Honolulu, HI 96818 USA; 3 National Marine Fisheries Service, Southeast Fisheries Science Center, 75 Virginia Beach Drive, Miami, FL 33149 USA

**Keywords:** Marine biology, Behavioural ecology

## Abstract

Red snapper and gray triggerfish are ecologically, economically, and culturally important reef fishes in the northern Gulf of Mexico (nGOM). Scientists and managers have sought to understand the effects of artificial reefs on reef fish ecology by focusing on fish residency and movement at artificial reefs with less attention paid to broader spatial and temporal patterns in reef fish movements among a seascape of artificial reefs and other natural habitats. We used novel large-scale (> 15 km^2^) geopositioning acoustic telemetry arrays to track the 3-dimensional movements of tagged red snapper (n = 59) and gray triggerfish (n = 15) among multiple nGOM artificial reefs up to 333 days. Tagged fish moved frequently among artificial reefs and had shorter residence times at the release reef (43 days for red snapper and 3 days for gray triggerfish) than reported in previous studies. Both species displayed high individual variability in movement dynamics, as well as seasonally variable diel patterns of habitat use, height above bottom, and distance to reefs, which may have been driven by dynamic influences of predation risk, physiological constraints, or foraging over time and space. The wider seascape view revealed in this study demonstrates the importance of including multiple artificial reefs over long timescales to capture individual, spatial, and temporal variability in reef fish movement.

## Introduction

Underwater acoustic telemetry enables a deeper understanding of fish movement ecology, including habitat use and selection^1–3^, diel and seasonal patterns of fish movement^4,5^, influence of environmental conditions and storms on broadscale movement^6,7^, and individual variability of movement and behavior^1,2,8^. In acoustic telemetry studies, fish are tagged with relatively small (< 5% of body weight) electronic tags, either surgically implanted within the body cavity or attached externally, which transmit unique ultrasonic codes to stationary underwater data-logging hydrophones. Advanced geopositioning acoustic telemetry relies on time-difference-of-arrival data processing of acoustic detection data to provide near-continuous fine-scale (within several meters) position estimates of tagged animals^9–12^. Acoustic telemetry studies that combine habitat information with continuous geoposition estimation over spatial and temporal scales that are ecologically relevant to the species and life stages being studied are by far the most valuable for understanding spatial ecology^1,3,13^. Ongoing technological advancements in acoustic telemetry technology, improved techniques for array deployment and retrieval^14^, and cooperation among networks of acoustic telemetry researchers^15,16^ have greatly increased our ability to track fish movement in open ocean marine habitats.

Geopositioning acoustic telemetry deployed on large temporal (months to years) and spatial (10 s of km) scales provides unique opportunities to study movement dynamics of marine fishes in situ. These large datasets enable exploration of some broader concepts in spatial ecology that likely drive individual movements, including foraging arena theory, landscape ecology, resource partitioning, and temperament-dependent behaviors. Foraging arena theory suggests the survival of individual fishes, and by extension population-scale vital rates, are determined by trophic interactions between predators and prey that occur over limited time and space in the ‘foraging arena’^17,18^. Marine fish often feed on diverse prey items throughout the year^19–21^, indicating the community composition of foraging arenas shifts seasonally. Attributes defining the foraging arenas of marine fishes may shift over space and time, and may be observable via the movements of individuals^22,23^. Foraging arenas likely exist within a mosaic landscape of patches characterized by distinct habitats and ecological communities^24^. The movements of individual fish among estuarine and marine ‘seascapes’ have been studied in recent years using acoustic telemetry^1,3,13,25^. Within the seascape, spatially and temporally variable habitats and their associated biological communities constitute the dimensions over which species partition resources and ecological niches develop^26^. Acoustic telemetry studies providing concurrent detailed movement observations for multiple species have contributed evidence to infer resource and habitat partitioning among sympatric fish species^27,28^.

In addition to diversity among species, individuals within populations may show great variability in partitioning of resources and evaluating the risk-reward trade-offs of visiting the foraging arena as demonstrated by movements and behavior^1,23,29,30^. Movement across the seascape and resource partitioning may vary among individuals according to variables such as size or ontogeny^31^ or sex^13^, but may also be driven by sets of behavioral traits (a temperament) that remain consistent across time and situations^32–34^. For example, individuals displaying active (“bold”, “fast”, “risk-prone”, or “mover”) temperaments may be more aggressive towards prey and conspecifics, less cautious of predators, farther ranging, and more likely to move among patchy habitats compared to their passive (“shy”, “slow”, or “stayer”) counterparts^33,35,36^. Temperament, as a characteristic of within-species variation, provides a link to evolution via differential selection^37^. Further application of high accuracy, extended duration, and large spatial extent acoustic telemetry to study movements of multiple fish species concurrently across the seascape are likely to reveal additional insights in these broad spatial ecology concepts^38,39^.

Reef fishes on the U.S. Gulf of Mexico continental shelf, including red snapper (*Lutjanus campechanus*) and gray triggerfish (*Balistes capriscus*), support valuable commercial and recreational fisheries^40^. In U.S. waters of the northern Gulf of Mexico (nGOM), red snapper and gray triggerfish are often caught by recreational fishers at artificial reefs due to these species’ general tendency to aggregate at high densities around patchy structured habitats located in otherwise open substrates^41,42^. Artificial reefs include oil and gas infrastructure, shipwrecks, construction debris, and structures constructed of cement and other materials purpose-designed to attract reef fish. Artificial reefs are a classic example of spot disturbance patches within a seascape^24^ and host the foraging arenas where fishermen and reef fish interact. The effects of artificial reefs on nGOM reef fishes at levels of the community^41,43^, population^42,44,45^, and individual biology^19,46–49^ have been the focus of research and debate for many years. A primary focus of many studies in the nGOM, particularly of red snapper, has been on demonstrating the relative importance of artificial reefs by quantifying the extent of movement of adult fish away from artificial reef sites. A paradigm has emerged suggesting nGOM reef fish, including red snapper and gray triggerfish, display high fidelity to individual artificial reefs^5,50–52^. However, artificial reefs in the nGOM are not isolated habitat units, but instead create a 3-dimensional mosaic seascape of potential refuges and foraging arenas for reef fish. Observing fish movement on a seascape scale provides a wider view of fish movements away from any single artificial reef and presents the opportunity to study potential drivers of individual movement among multiple artificial reefs.

**Figure 1:**
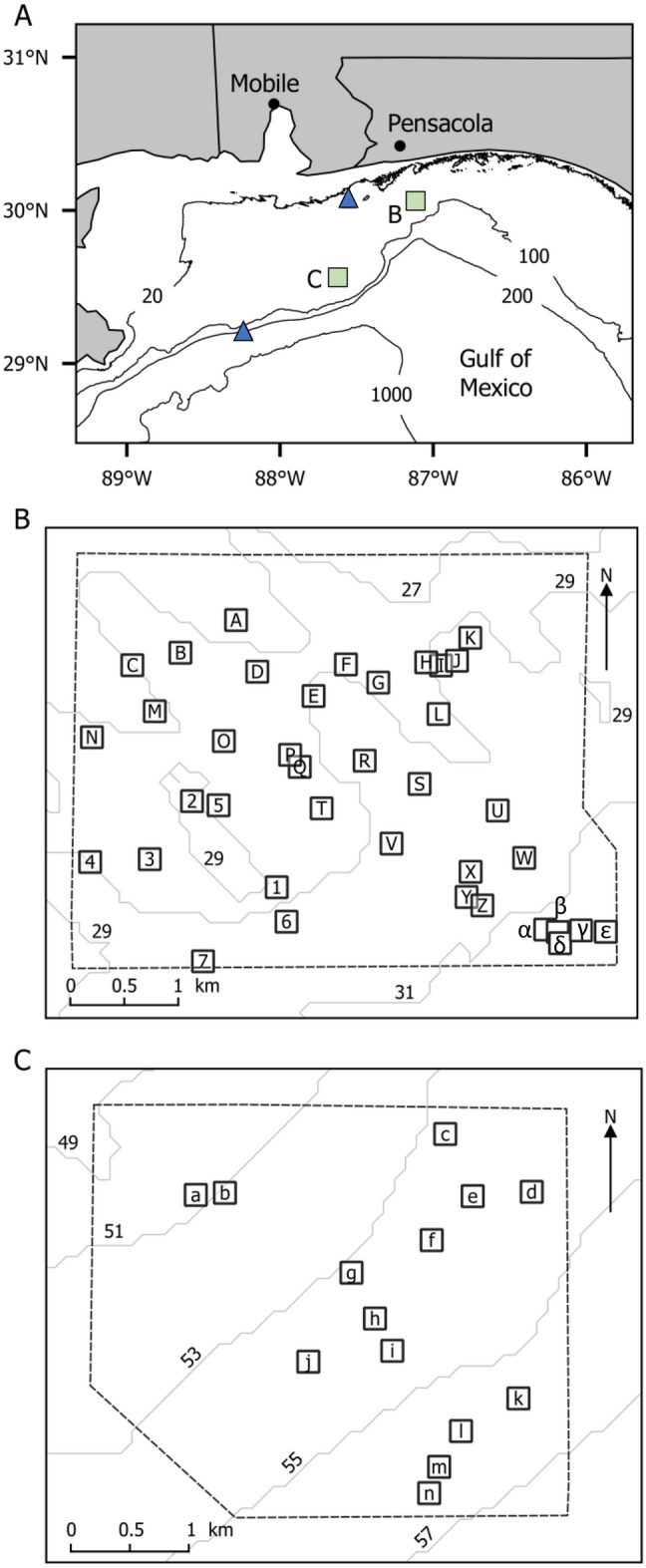
Map of the study area. (**A**) Location of acoustic telemetry arrays (squares) and oceanographic data buoys (triangles) in the northern GOM. Depth contours are labeled in meters. Details are shown for the (**B**) 30-m and (**C**) 55-m acoustic telemetry arrays. The approximate positioning boundaries of each array are noted by a broken line, artificial reefs are noted with labeled squares, and depth contours are labeled in meters.

In this study, we used geopositioning acoustic telemetry to track the 3-dimensional movements of tagged red snapper and gray triggerfish at two 15-km^2^ study sites of open bottom and artificial reef habitat at two depths (30 and 55 m) in the nGOM (Fig. 1). Our objectives were to (1) characterize movements of fish across a mosaic seascape of artificial reefs and natural bottom; (2) quantify the 2-dimensional area and the 3-dimensional volume of habitat used by tagged fish; (3) describe the physical association of tagged fish to structural habitat features of the seascape, including the sea floor and artificial reefs; (4) quantify how environmental, temporal, and individual variables relate to fish movement and behavior; and (5) investigate whether individuals display heterogeneity in movement and behavior indicative of bold (“mover”) and shy (“stayer”) temperaments. Study results allow hypothesis testing and inference about red snapper and gray triggerfish movement dynamics that was previously not possible. Implications for their ecologies, fishery exploitation, and fisheries management are discussed.

## Results

**Figure 2:**
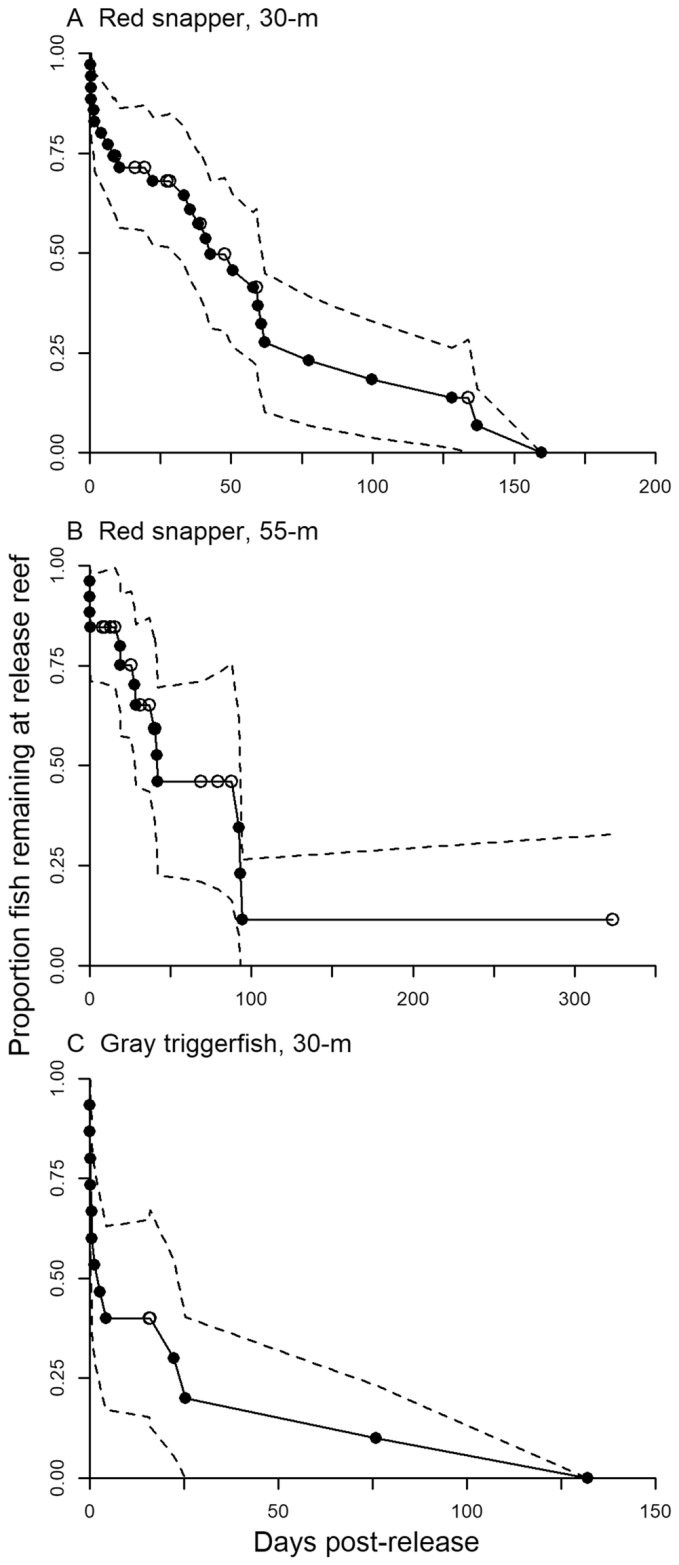
Kaplan–Meier estimated (with 95% CI) release reef residency of red snapper in the (**A**) 30-m array and (**B**) 55-m array, and (**C**) gray triggerfish in the 30-m array. Filled symbols mark when tagged fish moved away from the release reef and open symbols represent right censored individuals.

**Figure 3:**
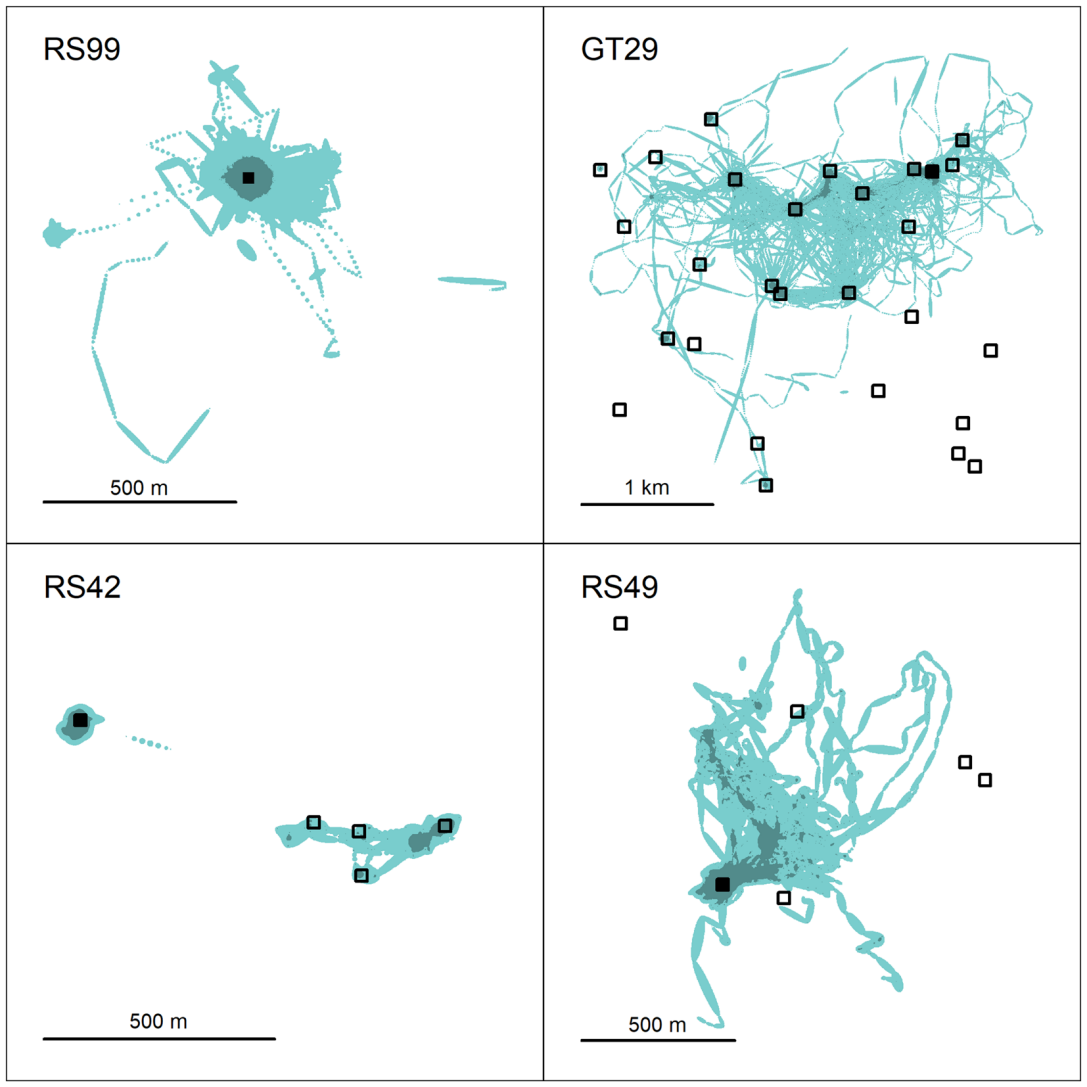
Brownian Bridge modeled area used by 4 tagged fish over the duration of the study: red snapper RS99, tracked 236 days in the 55-m array; gray triggerfish GT29, tracked 266 days in the 30-m array; red snapper RS42, tracked 144 days in the 30-m array; and red snapper RS49, tracked 123 days in the 30-m array. Artificial reefs are marked with square symbols, while the reef where each fish was tagged and released is filled. For each figure, darker shading represents the home range (95% KDE) and lighter shading represents all modeled area used over the entire study period.

Thirty-four (34) red snapper (mean total length, TL = 404 mm, range 331–625 mm) and 15 gray triggerfish (mean fork length, FL = 426 mm, range 330–486 mm) were present within the 30-m depth array for between 4 and 333 days (Supplementary Tables 1 and 2). Twenty-five (25) red snapper (mean TL = 548 mm, range 412–761 mm) were present within the 55-m depth array for between 4 and 324 days. Median residency times at the release reef were 43 days (95% CI 33–62 days) for red snapper in the 30-m array, 42 days (95% CI 29–93 days) for red snapper in the 55-m array, and 3 days (95% CI 1–25 days) for gray triggerfish in the 30-m array (Fig. 2). Reef switching behavior was highly variable among individuals. For example, RS99 remained near the tagging reef for the duration of the study (324 days) while RS42 visited 5 reefs over the 144 days it was tracked (Fig. 3). Gray triggerfish generally visited a greater number of reefs and switched between them more frequently than did red snapper. For example, GT29 visited 18 different reefs, switching between reefs 625 times over 266 days.

Median daily home range was 1890 m^2^ (23,800 m^3^) for red snapper in the 30-m array, 1780 m^2^ (37,600 m^3^) for red snapper in the 55-m array, and 2770 m^2^ (35,100 m^3^) for gray triggerfish in the 30-m array. Similar to reef switching behavior, home range was also highly variable among fish. For example, RS42’s median daily home range was 680 m^2^ (8400 m^3^), even though this fish made occasional movements between neighboring reefs (Fig. 3). In contrast, RS49 only used 2 reefs during the study period but made numerous trips up to 1 km away from the reef where it was tagged and had a larger median daily home range of 2630 m^2^ (37,500 m^3^, Fig. 3). Movement, home range, and reef residence summaries are included for all fish in the Supplementary Materials (Supplementary Tables 1 and 2; Supplementary Figs. 1–5).

Oceanographic conditions in the areas of both arrays were generally calm during the study period. In the 30-m array, the upper 95% quantile of observations of sustained wind speed, peak wind speed, and wave height were 10.1 m/s, 12.2 m/s, and 1.62 m, respectively (Supplementary Fig. 6). The upper 95% quantile of observations of sustained wind speed, peak wind speed, and wave height in the 55-m array were 10.6 m/s, 13.6 m/s, and 2.30 m, respectively (Supplementary Fig. 7). Bottom water temperature was generally warmer in the 30-m array (range 18.7–30.0 °C) than in the 55-m array (range 16.1–27.8 °C). Storm systems caused high winds (peak gusts to 20.3 m/s in the 30-m array and 25.9 m/s in the 55-m array) and large waves (to 3.3 m in the 30-m array and 9.2 m in the 55-m array) sporadically throughout the study.

**Figure 4:**
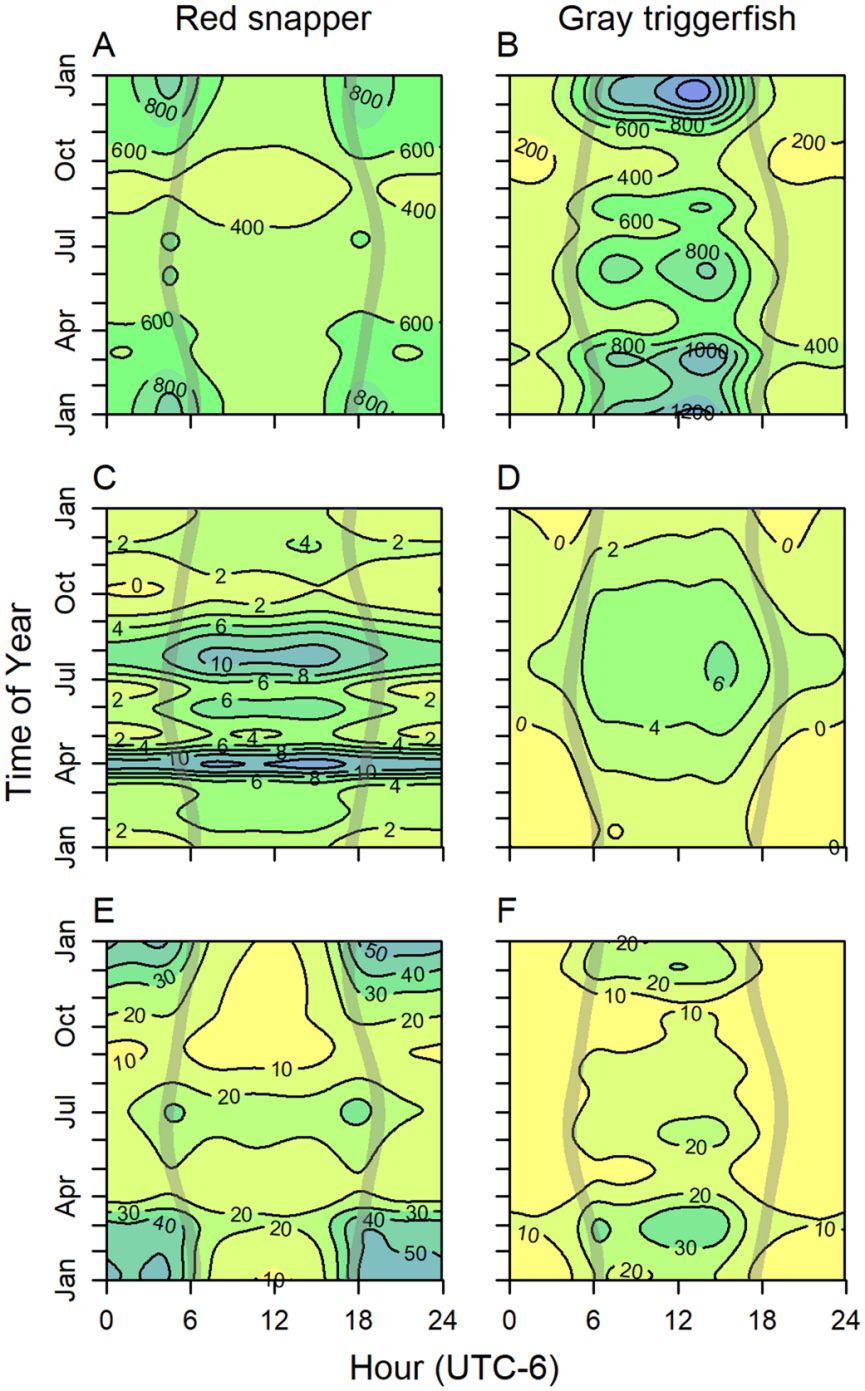
GAMM predicted hourly home range (m^2^; **A**,**B**), height above bottom (m, **C**,**D**), and distance to reef (m, **E**,**F**) of red snapper and gray triggerfish as a function of time of day and time of year. Overshaded areas represent dawn and dusk.

**Figure 5:**
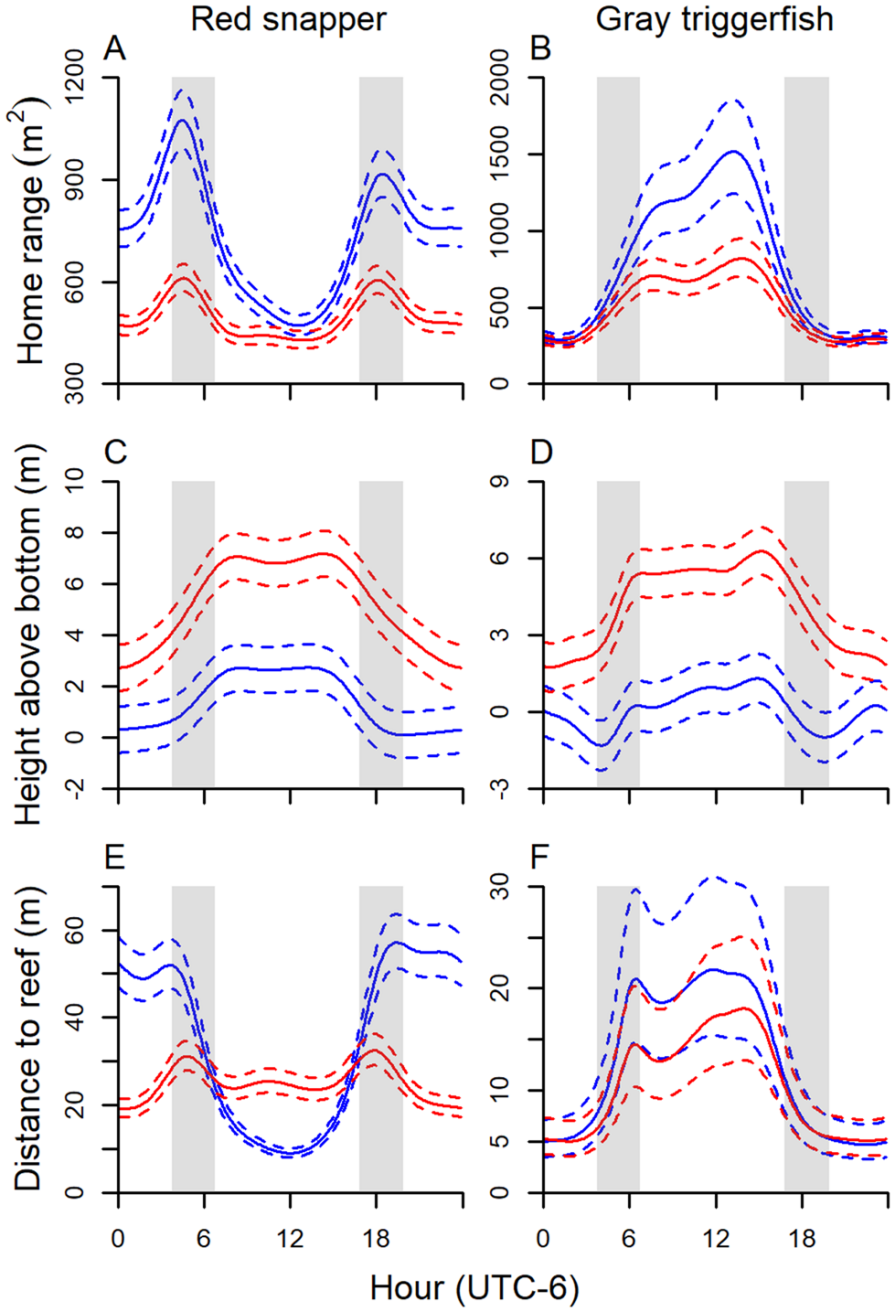
GAMM predicted (solid line) and 95% CIs (broken line) of hourly home range (95% KDE; **A**,**B**), height above bottom (**C**,**D**), and distance to reef (**E**,**F**) of red snapper and gray triggerfish as a function of time of day. In each figure, January 1 is blue, July 1 is red. Overshaded areas represent the maximum dawn and dusk periods over the whole year.

**Figure 6:**
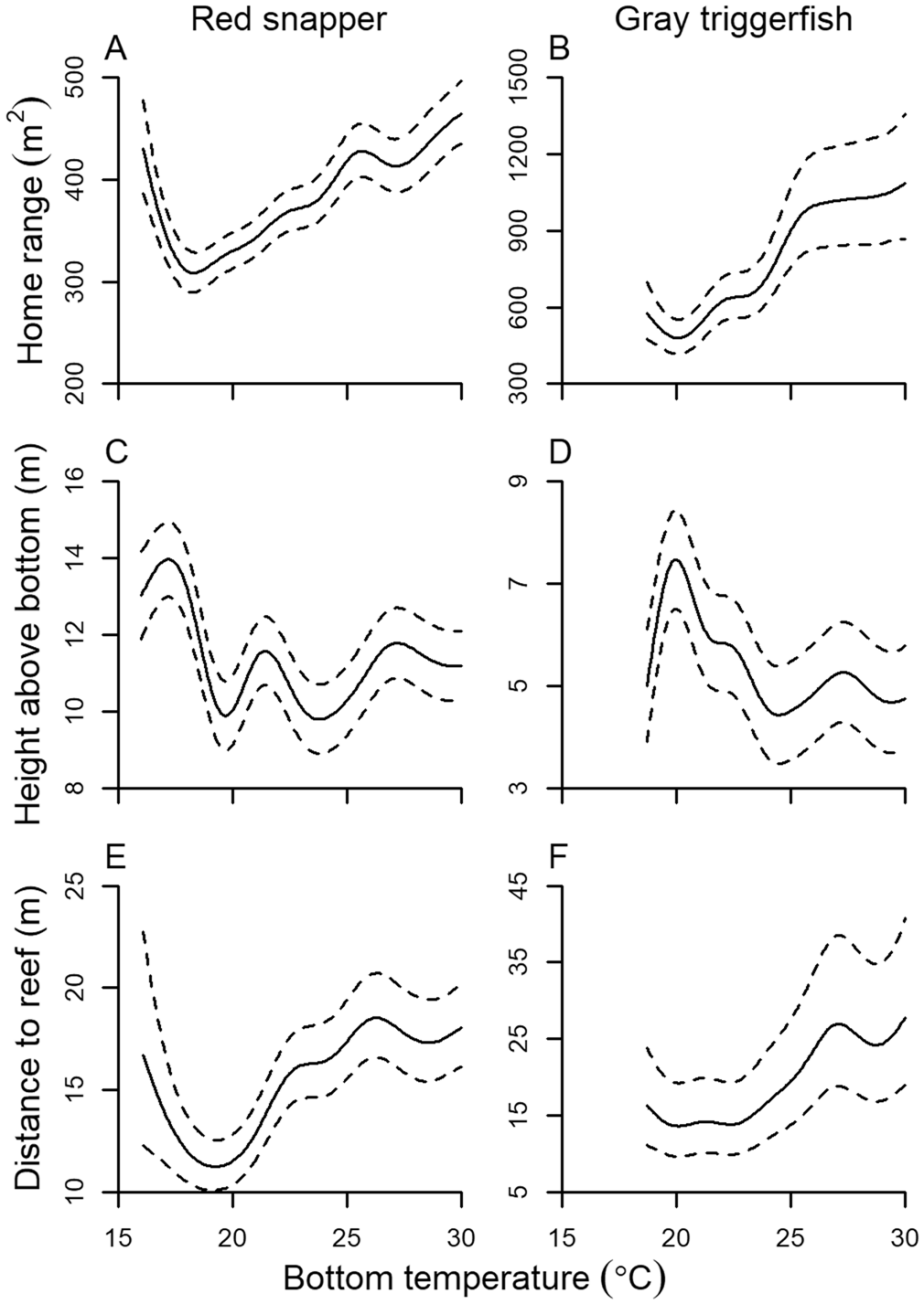
GAMM predicted (solid line) and 95% CIs (broken line) of hourly home range (95% KDE; **A**,**B**), height above bottom (**C**,**D**), and distance to reef (**E**,**F**) of red snapper and gray triggerfish as a function of bottom water temperature.

The majority of environmental covariates was retained within the most parsimonious generalized additive mixed-effects models (GAMMs) for red snapper and gray triggerfish home range, height above bottom, and distance to reef. Results were similar for GAMM predicted home range in both 2- and 3-dimensions, therefore, unless otherwise noted, results are discussed for 2-dimensional home range. Models described between 12 and 26% of variance in the data (Table 1). Smoothing terms for hour, day, bottom temperature, and the interactions between hour × day were generally most informative in the models (Supplementary Tables 3 and 4). Red snapper displayed a clear crepuscular pattern in home range; they were most active during crepuscular (dawn and dusk) periods, and the pattern was most pronounced in the winter months from November to February (Figs. 4A, 5A). In contrast, gray triggerfish were most active during the daytime and minimally active at night (Figs. 4B, 5B). Similar to red snapper, variation by hour in home range for gray triggerfish was strongest during winter months. Both species were generally higher in the water during the day and during summer months (Figs. 4C,D, 5C–D, Supplementary Fig. 8). Gray triggerfish typically stayed within several meters of the seabed year-round while red snapper displayed a more pronounced seasonal trend and were generally higher in the water column, moving from close to the bottom (generally less than 4 m) during the fall and winter to 10 + m above the seabed during spring and summer. Red snapper displayed two distinct daily patterns of distance from the reef (Figs. 4E, 5E). From April through September, red snapper generally stayed within 30 m of the reef and moved farthest from the reef during crepuscular periods. In contrast, from October through March, fish stayed close to the reef during the day and moved 50 + m away from the reef at night. Gray triggerfish daily patterns in distance to reef were consistent throughout the year, moving farthest away during daytime and closest at nighttime, with the largest diurnal versus nocturnal differences occurring during December to March (Figs. 4F, 5F). Both species were increasingly active and moved farther from the reef as bottom water temperature increased above 20 °C (Fig. 6). However, the trend reversed and red snapper displayed larger home range and moved farther away from reefs when water temperature was lowest (e.g., below 18 °C, which occurred only in the 55-m array during the 2017 hurricane season).

**Table 1:** Significance of GAMM components describing red snapper and gray triggerfish hourly 2-dimensional home range, height above bottom, and distance to reef. Random intercepts were included a priori for individual (both species) and array (red snapper only). Missing values indicate the model component was not included in the most parsimonious model.

Model component	Red snapper			Gray triggerfish		
	Home range (r^2^ = 0.12)	Height above bottom (r^2^ = 0.17)	Distance to reef (r^2^ = 0.17)	Home range (r^2^ = 0.26)	Height above bottom (r^2^ = 0.17)	Distance to reef (r^2^ = 0.17)
**Fixed effects**						
Intercept	< 0.001	< 0.001	< 0.001	< 0.001	< 0.001	< 0.001
Wave height	0.921	0.676	0.122	< 0.001	0.269	< 0.001
Wind speed, sustained		0.096			0.622	0.429
Wind speed, gust	0.096		0.004			
Atm. pressure	0.501	< 0.001	0.034	0.468	0.843	0.272
Temperature	< 0.001	< 0.001	0.070	0.103	< 0.001	0.811
Fish length	< 0.001			0.013		
**Smooth terms**						
Hour	< 0.001	< 0.001	< 0.001	< 0.001	< 0.001	< 0.001
Day	< 0.001	< 0.001	< 0.001	< 0.001	< 0.001	< 0.001
Day × hour	< 0.001	< 0.001	< 0.001	< 0.001	< 0.001	< 0.001
Moon phase		< 0.001	< 0.001		< 0.001	< 0.001
Moon phase × hour	< 0.001	< 0.001	< 0.001	< 0.001	< 0.001	< 0.001
Wave height	< 0.001	< 0.001	< 0.001	< 0.001	< 0.001	< 0.001
Wind speed, sustained		< 0.001			< 0.001	< 0.001
Wind speed, gust	< 0.001		< 0.001			
Wind direction		< 0.001	< 0.001	< 0.001	< 0.001	< 0.001
Atm. pressure	< 0.001	< 0.001	< 0.001	< 0.001	< 0.001	< 0.001
Temperature	< 0.001	< 0.001	< 0.001	< 0.001	< 0.001	< 0.001
Fish length	< 0.001			0.004

**Figure 7:**
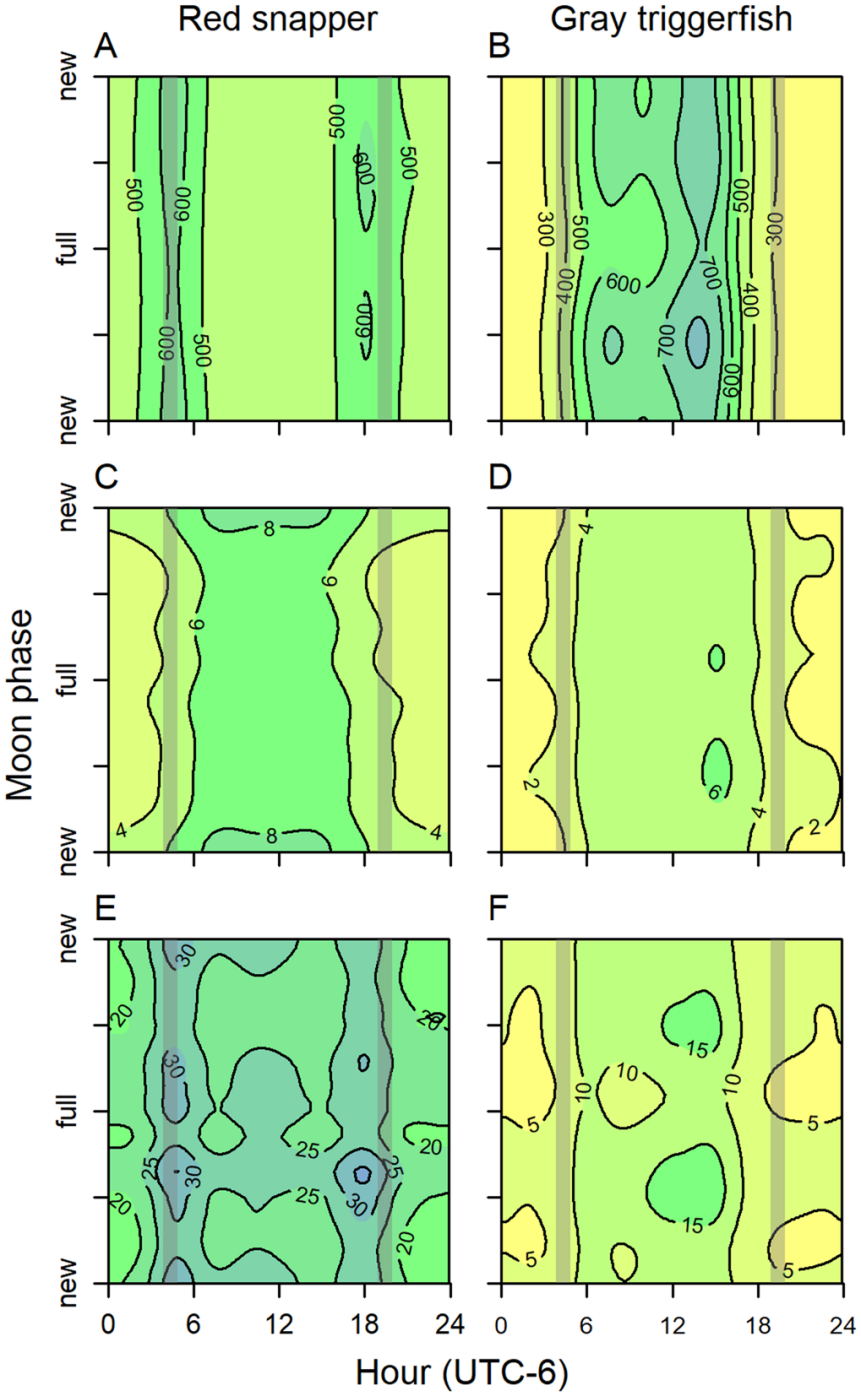
GAMM predicted hourly home range (m^2^; **A**,**B**), height above bottom (m, **C**,**D**), and distance to reef (m, **E**,**F**) of red snapper and gray triggerfish as a function of time of day and moon phase, for July 1. Overshaded areas represent dawn and dusk.

Although significant as a random smoothing term in all models, the effect of moon phase (either alone or its interaction with hour or time of year) on red snapper or gray triggerfish movement or height off bottom was not consistent between species (Fig. 7; Supplementary Fig. 9). For red snapper, home range was largest during dusk and the first and last quarter moons, and fish moved higher in the water during all times of day around the new moon. Gray triggerfish did not show differences in nighttime behavior according to moon phase, but home range, height off bottom, and distance to reef were all greatest during the early afternoon hours of the first quarter moon.

**Figure 8:**
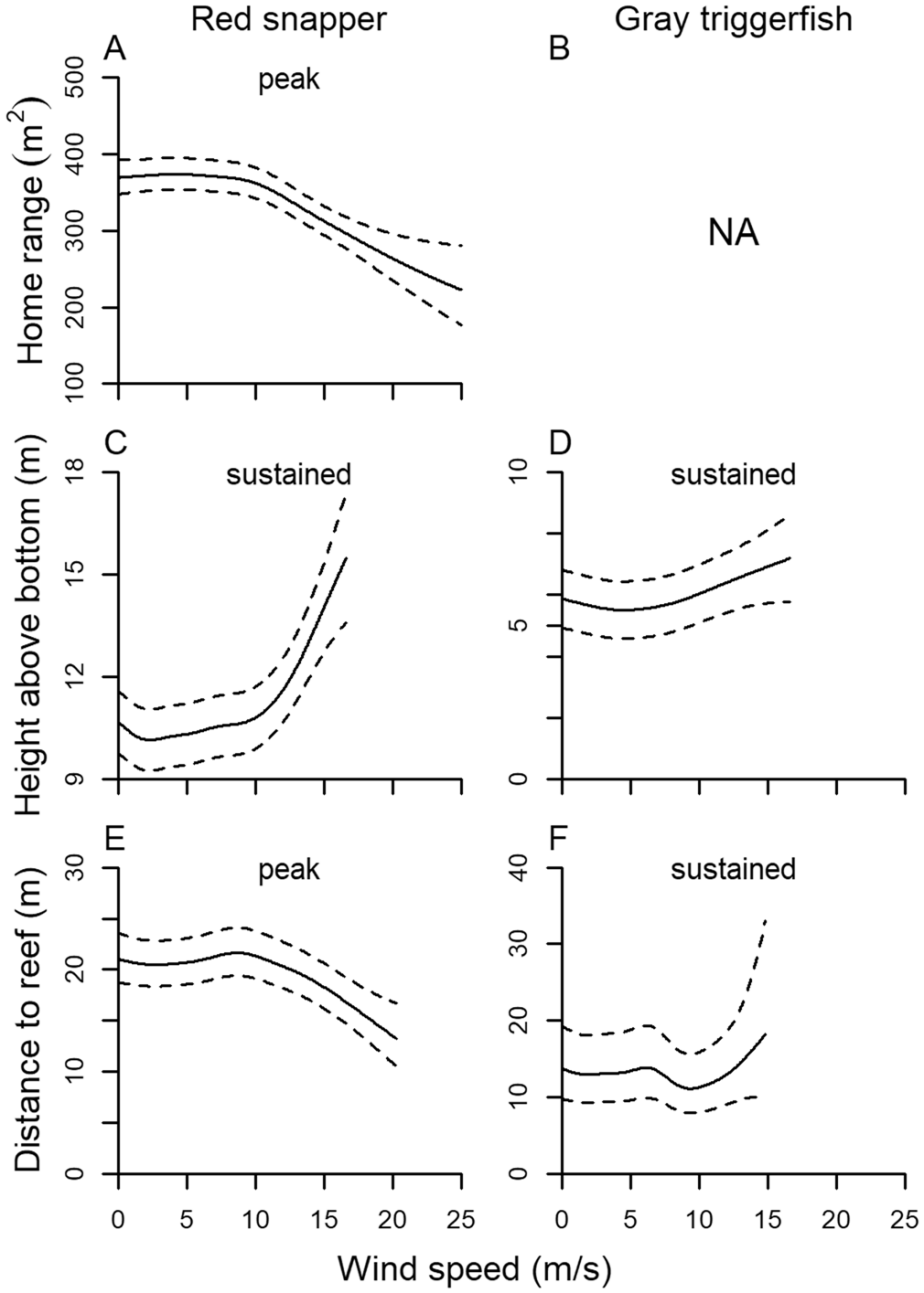
GAMM predicted (solid line) and 95% CIs (broken line) of hourly home range (95% KDE; **A**,**B**), height above bottom (**C**,**D**), and distance to reef (**E**,**F**) of red snapper and gray triggerfish as a function of sustained or peak wind speed.

Red snapper displayed smaller home ranges, moved higher in the water, and stayed closer to reefs when wind speed increased above 10 m/s (Fig. 8). As was observed for wave height, GAMM predicted trends in wind speed and home range, height above bottom, and distance to reef of tagged gray triggerfish were less apparent, possibly obscured by large fish movements during a few infrequent high wind and large wave events (Supplementary Figs. 10–13).

**Figure 9:**
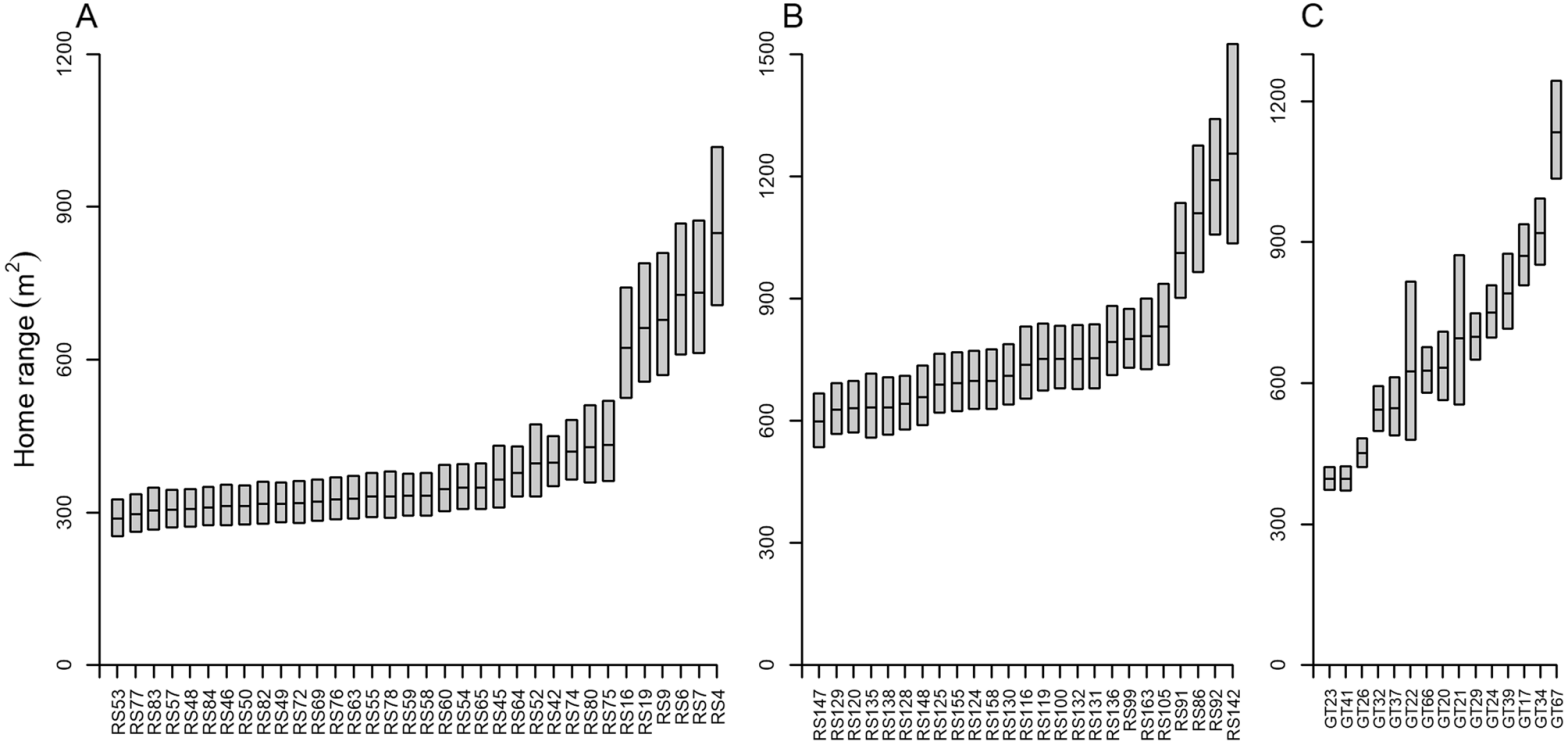
GAM predictions (with 95% CI) of hourly home range for individual (**A**) red snapper in the 30-m array, (**B**) red snapper in the 55-m array, and (**C**) gray triggerfish in the 30-m array. The best fit GAM model was modified to include Fish ID as a fixed, rather than random effect, and predictions were made based on the average conditions experienced by fish in each array, hence, (**A**) and (**B**) cannot be directly compared.

Predicted hourly home range varied among individuals when all other factors were controlled for in the selected GAMs, as revealed by non-overlapping 95% confidence intervals predicted by the refitted models with individuals as a fixed effect (Fig. 9). Eighteen percent (6 of 34) of red snapper in the 30-m array and 16% (4 of 25) of red snapper in the 55-m array were more active and had notably larger hourly predicted home ranges than other fish. Home ranges of gray triggerfish were also highly variable within and among individuals. Three (3) gray triggerfish (20% of 15 tagged fish) had smaller home ranges and 1 Gy triggerfish (7% of 15 tagged fish) had a much larger home range than other tagged fish.

Several tagged gray triggerfish displayed periodic behavior patterns during the summer. Of the 7 tagged gray triggerfish in this study that were within the array during June–August, 3 exhibited periods of relative inactivity and near-zero height above bottom that commenced within 2 h of sunrise (05:00–08:00 local time) and persisted for 35–48 h. GT34 displayed 4 events, separated by intervals of 12, 16, and 34 days (Supplementary Fig. 16). We observed only 1 event of similar inactivity each for GT23 and GT29.

## Discussion

Study findings do not support the hypothesis that nGOM reef fish such as red snapper or gray triggerfish display high fidelity to individual artificial reefs. We estimated median site residency of red snapper (42–43 days) to be much lower than from mark-recapture studies (122–387 days)^45,52–55^ and several acoustic telemetry studies (202–700 days)^5,51,56,57^. For gray triggerfish, the disagreement between our findings and previous studies is even more substantial. Median site residency of the gray triggerfish in this study was 3 days (95% CI 1–25 days) in contrast to previous estimates ranging from 223 to 567 days^50,53,55^. However, to our knowledge, there have been no other positioning acoustic telemetry arrays deployed on this scale to track movement of nGOM reef fishes, and our findings that red snapper and gray triggerfish move often among reefs may be due largely to the greater resolution afforded by the spatial scale of our arrays. For instance, mark-recapture studies cannot account for the movements of fish between mark and recapture, and may also be affected by positive observation bias at the release site if release reefs are frequently re-visited and fished by fishermen or researchers throughout the study^54^. In acoustic telemetry studies where fish are tracked in detail over only a limited area around the release site^5,57^, tagged fish moving away from the release reef to visit adjacent reefs before returning may be misidentified as “resident” on the release reef. Each of our deployed acoustic telemetry arrays covered a 15-km^2^ area and encompassed numerous (14–38) artificial reefs of diverse types (ranging in size, shape, and material), and we observed fish moving frequently, sometimes daily, among many (up to 18) artificial reefs. Our results suggest increases in acoustic array coverage area, particularly for fine-scale positioning studies, may further elucidate metrics of fish movement, reef use, site fidelity, and spatial ecology across the broader habitat seascape, shifting the emphasis away from presence/absence observations relative to reefs where fish are originally captured, tagged, and released.

Characteristics of our study sites within the nGOM seascape, including the density and diversity of artificial reefs, may have been conducive to reef switching behavior while allowing fish to use smaller home ranges over shorter time periods, relative to tagged fish in other studies. On an hourly time-scale, we estimated home range of red snapper (< 1000 m^2^) and gray triggerfish (< 1500 m^2^) to be less than estimates from previous studies in this region^5,50^, possibly due to the scale and configuration of acoustic arrays used, as discussed above. A recent study in the western GOM^4^ provided movement information on red snapper over high density artificial habitat. Estimated mean daily home range (95% KDE) of tagged fish was approximately 78,000 m^2^, which is over a magnitude greater than estimates we report here for red snapper (Supplementary Tables 1 and 2). The authors suggested the high density of artificial reefs in the western GOM study may have placed heavy pressure on prey resources and elevated competition to the level that fish were forced to range over a large area. In the current study, average distance between nearest neighboring artificial reefs was 350 m in the 30-m array and 520 m in the 55-m array. It is possible the intermediate density of artificial reefs in our study area, combined with the greater diversity of artificial reef types, encouraged sporadic reef switching by fish foraging on diverse prey items, but also afforded lower levels of resource competition among conspecifics, thus allowing fish to use smaller areas at shorter times scales. This heightened efficiency of space use, especially for red snapper, suggests the diversity and density of artificial reefs in our study site may be a favorable choice for managers interested in establishing new areas of artificial reefs in the nGOM.

We found time of day and time of year were significant drivers of reef fish home range, height above bottom, and distance to reef. Moreover, there were significant interactions between time of day and time of year, suggesting diel patterns in fish behavior shift according to season. Seasonal shifts in diel movement patterns were often similar among conspecifics but differed between red snapper and gray triggerfish, which suggests resource partitioning between the two species. We believe the distinct physiology and ecology of the study species interacting with the trade-offs between obtaining food resources and avoiding predation were the primary drivers of general movement and space use. High variability in red snapper seasonal movement patterns may be the result of prey switching behaviors throughout the year as red snapper are highly opportunistic, generalist feeders^19,58^. During the winter (November–February), red snapper displayed a strong peak in activity (maximum hourly home range) at dawn and dusk, were consistently within several meters of the bottom, and moved greatest distances from the reef at nighttime (40–60 m, contrasted to < 20 m during the daytime). This movement pattern suggests fish were actively feeding over large areas of bottom from dusk to dawn, likely on sand-dwelling demersal organisms such as decapod crustaceans, in agreement with diet and stable isotope analyses suggesting red snapper feed mostly on benthic organisms during winter months^20^. Red snapper movement patterns shifted markedly during late March and early April when crepuscular peaks in activity and nightly movements away from reefs ceased. Instead, tagged fish remained close to reefs (within 20 m) but moved higher in the water column (10–12 m above the seabed at night and 12 + m during the day). Results of several studies based on both stomach content and stable isotope analyses have established that adult red snapper at artificial reefs in the nGOM subsidize their diet with pelagic zooplankton, particularly pteropods^46,58^ during spring and summer^19,20,59^. It seems likely, then, that red snapper moved higher in the water column to forage on plankton in spring and summer. Generally, we found red snapper were most active during nighttime and winter months, however, authors of several acoustic telemetry investigations have concluded red snapper are most active during daylight hours and warmer summer/fall months^60,61^ with noted variations among study sites^5^ and individuals^51,62^. Wide variation in red snapper movement and behavior seasonally, likely in response to shifting availability of food resources and variable predation pressure, suggests movement patterns may be driven by a confluence of habitat structure, environmental conditions, and community composition. In addition to different methodology and characteristics of the study site, difficulty in comparing this study to previous investigations may be caused by shifting behaviors and movement patterns over the lifetime of an individual (55 years for red snapper^63^ and 14 years for gray triggerfish^64^). As such, it is likely the limited timeframe of observation in this study (324 days for red snapper and 333 days for gray triggerfish) and previous studies (range 2–1096 days^5,62^) may be providing observations during dissimilar times within an individual’s lifetime, which may also increase apparent within-species variability of movement patterns.

Gray triggerfish displayed markedly different diel and seasonal movement dynamics than red snapper, likely because gray triggerfish are subject to different physiological constraints and feed on different prey items, as would be associated with resource partitioning among coexisting species^26,27^. Gray triggerfish have specialized dentition, including strong jaws and heavy teeth, and are known to consume large amounts of hard-shelled sessile sand- and reef- dwelling invertebrates, including echinoderms, barnacles, and bivalves^21,49,65^ that red snapper are not well equipped to handle. Movement patterns of gray triggerfish were more consistent throughout the year, suggesting individuals tagged in this study did not exploit seasonally abundant prey to the same extent as red snapper did, which is consistent with being more of a diet specialist. For example, gray triggerfish did not display the marked increase in height off bottom during spring zooplankton blooms, even though gray triggerfish are known to feed on pelagic prey items, including pteropods^21^. Both species may experience scarcity of food resources during the winter, thus foraging areas must be larger to acquire enough food to meet their bioenergetic demands. Activity, height above bottom, and movement away from reefs by gray triggerfish were consistently lowest at night while peak activity, height above bottom, and distance to reef occurred during daylight hours. This general movement pattern of low activity during the night and high activity during the day has been observed in previous studies^2,7,50^ and may suggest gray triggerfish depend heavily on vision to find prey or avoid predation, increasing the risk to reward trade-off that gray triggerfish would face venturing beyond several meters away from reefs in low light conditions^30^.

Artificial reefs may provide refuge to reef fishes from large predators, so individuals that are less active, closer to the bottom, or closer to the reef would likely be less vulnerable to predation, while more active fish that move higher in the water or farther away from the reef are more vulnerable to predation. During late summer and fall, red snapper and gray triggerfish home range and distance to reef reached a minimum (generally < 400–600 m^2^ per hour and < 20 m from reefs). Several species of sharks, including tiger (*Galeocerdo cuvier*), sandbar (*Carcharhinus plumbeus*), and sharpnose (*Rhizoprionodon terraenovae*), are known to prey on red snapper, gray triggerfish, and other reef fishes^66–70^. Seasonal peaks in shark occurrence^71^, perhaps driven by inshore-offshore or north–south migrations of large sharks^16,72^, may increase predation pressure on red snapper and gray triggerfish in late summer and fall. During periods of high predation pressure, red snapper especially may alter foraging patterns to avoid actively foraging over large areas. Instead, depth and distance to reef patterns agree with findings of previous studies that red snapper are opportunistically feeding on pelagic organisms above the reef, especially during daytime^46^, while venturing short (< 50 m) distances away from the reef to forage for sand-dwelling invertebrates during crepuscular periods^73^.

Aside from foraging and predation avoidance, spawning-associated behaviors may have also affected observed variability of movement patterns among individuals and over time. Based on length, all red snapper and gray triggerfish in this study were likely sexually mature^48,74–76^. Similar to many nGOM reef fishes, both red snapper and gray triggerfish are batch spawners with protracted summer spawning seasons, peaking in June–July^48,77,78^. Red snapper are broadcast spawners of pelagic eggs and would be expected to move higher in the water column to release and fertilize eggs every 2.5–6.5 days, with larger females spawning more frequently over the longest part of the season^48,77,79^. Although tagged red snapper likely engaged in spawning activity during this study, we do not believe spawning was a prominent driver of the general movement patterns we observed. Jackson et al. concluded, based on histological examination of gonads, that red snapper in the nGOM spawn during the afternoon hours (1330–1830). We observed red snapper higher in the water column during all daylight hours (0600–1800), so unless fish begin to aggregate 6–12 h before spawning, initial movement off the bottom would not likely be motivated by spawning. Also, examination of individual red snapper height above bottom does not reveal any multiple-day periodicity. Instead, red snapper move higher in the water every day, indicating they are likely responding to a daily occurrence in the environment, such as foraging opportunities.

Gray triggerfish are demersal nest-builders whereby a dominant male guards several nests around the reef that are each closely attended by a female with the eggs hatching 24–48 h after fertilization^80,81^. Beginning in May–June, dominant male gray triggerfish may reduce movement among reefs and spend much time excavating nest sites and patrolling within 15 m of the reef, while spawning females may reduce foraging activity and tend to nests^80^. Taken together, these behaviors of both males and females may have contributed to the relative reduction in activity and reduced distance to reef shown by gray triggerfish during the summer, contrary to the expected positive influence of increasing water temperatures on fish movement^50^. We identified several individual gray triggerfish (3 of 7 tagged individuals in the array June–July; Supplementary Fig. 16) that may have been females guarding nests. Histological studies to determine fecundity and spawning frequency of gray triggerfish are challenging because females are rarely captured close to the time of spawning^78^. Although the small sample size and unknown sexes of tagged gray triggerfish in this study preclude robust conclusions regarding their spawning behaviors, acoustic telemetry studies of individual movements during the spawning season may provide a valuable and previously underutilized method to study gray triggerfish reproductive biology.

Our results are consistent with those of previous studies that suggested strong storms, including tropical cyclones, can increase movement of red snapper and gray triggerfish^7,53,55^. However, in the current study we focused on quantifying the effects of oceanographic conditions, not the occurrence of named tropical cyclones, on fish movement. Relatively rare high-intensity weather events (wave height 2.3–9.2 m and peak wind speed 12.2–25.9 m/s) increased red snapper home range, gray triggerfish home range, and gray triggerfish distance to reef, but excluding movement data during periods when wave height or wind speed were in the upper 1 percentile, the trend was reversed and both species occupied smaller home ranges and moved closer to reefs as wave height increased. According to GAMM predictions, fish movement, particularly red snapper distance to reef, increased at extreme low atmospheric pressure, but otherwise the trend was unremarkable. Although large waves and high winds affected array sites sporadically over the course of the study, atmospheric pressure fell below 1000 mbar only twice: January 2017 in the 30-m array during an unnamed winter storm and October 2017 in the 55-m array during Hurricane Nate. Therefore, few data were available to fit the GAMM at low atmospheric pressures and resulting confidence intervals were wide. Higher bottom water temperatures were associated with increased activity and fish distance to reef, as previously observed for red snapper^4,5,60^ and gray triggerfish^50^. However, even when the effects of season were controlled in the GAMM, red snapper home range and distance to reef were predicted to increase at temperatures below approximately 18 °C. Bottom temperature and the effects of strong storms may be confounded in the GAMM because Hurricane Irma coincided with the lowest temperatures that we observed throughout the study period (16.1–17.0 °C during 16–17 September 2017) when the large storm system pushed cold, high salinity water across the 30-m array site. Furthermore, although we found evidence that wave height, wind speed, atmospheric pressure, and water temperature influenced home range, height above bottom, and distance to reef for tagged fish, the effects were much less pronounced than trends associated with seasonal or diel cycles, suggesting most movement behavior for red snapper is driven primarily by ecological constraints, not climatological phenomena.

Movement behavior was highly variable among individual tagged fish for both red snapper and gray triggerfish. Over hourly time scales, predicted home range by individual generally followed a continuum, displaying no obvious dichotomy of highly active vs. sedentary fish. However, over longer time scales (days to weeks), nearly 15% of tagged red snapper (7 of 34 in the 30-m array and 1 of 25 in the 55-m array) and roughly half of gray triggerfish (8 of 15 in the 30-m array) could be described as ‘super-switchers.’ These fish quickly traversed large (up to 1 km) distances of open sand to move to a different reef, on average more frequently than every 2 days. These red snapper super-switchers would more likely be recaptured away from their release site in a mark-recapture study, and may be synonymous with previously described movers^82^. Our finding that individual gray triggerfish more commonly exhibited active reef switching than red snapper is especially interesting given the conclusions expressed in previous studies that gray triggerfish were less likely than red snapper to be recaptured away from the release site when movements of the two species were compared within a single study^45,53,55^. It is possible gray triggerfish movements coincide with behaviors that limit their susceptibility to common recreational fishing gears (hooks baited with squid or fish). Individuals would be caught and tagged only at reefs where they are feeding in the water column above the structure. If tagged gray triggerfish travel to other reefs specifically to feed on hard-shelled benthic organisms or engage in non-feeding behaviors, then these fish movements would be invisible to conventional mark-recapture studies. This would be an example of differential catchability (and hence observability) of fish over time and space that is overcome with acoustic telemetry studies that better detect movement, regardless of the specific fish activity or movement pattern the fish.

Although we observed many highly active fish in our study, our observations are still likely skewed towards less active fish for several reasons. First, 32.2% of red snapper and 60% of gray triggerfish emigrated from the acoustic array between 4 and 269 days after release and were censored from this study at that time. Observations of these more active movers could not be made following their emigration, thus observations that were made throughout the duration of array deployments were skewed toward more sedentary fish. Secondly, red snapper movers have been observed more over natural hard-bottom than at artificial reefs^82^. To our knowledge, natural hard-bottom habitat was absent from the 30-m array and rare in the 55-m array, and all fish were caught, tagged, and released at artificial reefs. Finally, observing the true diversity of individual temperaments may require capturing and tagging fish from a large number of reef sites because individuals of similar phenotype may spatially aggregate in a non-random fashion^83^. Hence, the effective sample size available to investigate movement behavior would have been less than the number of individuals tagged because the number of artificial reefs where we captured and tagged fish was limited.

In summary, we provide metrics of fish movement and behavior that can be compared with future geopositioning acoustic telemetry studies (e.g., hourly and daily home range defined as 95% KDE, median site residency, number of reefs visited, height above bottom, and distance to reef). We found red snapper and gray triggerfish typically use substantial (> 1000 m^2^) areas encompassing a broad seascape of artificial reefs and natural habitat, as they frequently move great distances to transit between reefs. However, movement patterns and space use were highly variable among individuals, across space, and over time at both short (hourly and daily) as well as longer (seasonal) time scales, suggesting the ecological drivers of fish movement are dynamic. Given the variability of movement among species, individuals, space, and time, future benefits of deploying artificial reefs may be best realized not by focusing on quantity or area of reefs deployed, but by maximizing diversity in regard to reef type, density, and configuration. The increase of collaborative networks of acoustic telemetry researchers holds the promise that researchers could pool acoustic telemetry resources and tagging expertise to deploy larger positioning arrays and track multiple species and their interactions simultaneously^16^. In addition to improved understanding of fish movements over larger areas and timescales, acoustic telemetry studies should also be designed to investigate broader ecological principles in the marine environment, requiring multi-disciplinary collaboration among researchers. For example, full-coverage surveys of bottom habitat, estimates of fish density, and concurrent estimates of the abundance of prey resources and potential predators may provide a better understanding of the reef characteristics, density-dependent conditions, and predation risks within the foraging arena that may drive reef fish movement.

## Methods

### Acoustic telemetry array and fish tagging

An array of 60 Innovasea (formerly Vemco: Bedford, Nova Scotia, Canada) model VR2Tx and VR2W data-logging acoustic receivers was deployed in the northern GOM (Fig. 1A) from February 2016 to March 2017 at 28–35 m depths ("30-m" array; Fig. 1B, Supplementary Fig. 1); another array of 46 receivers was deployed from August 2017 to July 2018 at 48–55 m depths ("55-m array"; Fig. 1C, Supplementary Fig. 2). Receivers were mounted 2 m above the sea floor on poly-vinyl chloride (PVC) pipes embedded in 40-kg concrete bases that were retrieved with the assistance of a miniature ROV^14,70^. The receivers in each array encompassed approximately 15 km^2^ of predominantly sand and shell rubble flat-bottom habitat, interspersed with numerous single and clustered artificial reef structures. Artificial reefs ranged in relief from 1 to 4 m and were constructed from materials including cement, wood, iron rebar, and steel beams. The 55-m array may also have contained some small areas of low-relief (< 1 m) natural sedimentary rock ledge. The receivers in each array were placed in a grid with no receiver closer than approximately 10 m to an artificial reef. The locations of artificial reefs within the study area were determined based on knowledge of previous Florida Fish and Wildlife Conservation Commission (FWC) funded artificial reef constructions, local knowledge from fishermen, or partial coverage sidescan sonar surveys of the 30-m array provided by FWC between 2014 and 2019 (Anthony Knapp, pers. comm.). Our study areas also contained other artificial reefs that we were not aware of a priori, but we inferred their locations from estimated positions of many reef fish consistently centering on a location.

Red snapper (n = 141) and gray triggerfish (n = 26) were caught with hook and line at artificial reefs within each array, externally tagged^70^ with Vemco V13P-1 × pressure (depth) sensing acoustic transmitters, and released into the acoustic array during 4 tagging events: (1) spring 2016 in the 30-m array (26 April–3 May); (2) late summer 2016 in the 30-m array (14 September); (3) late summer 2017 in the 55-m array (2 September); and (4) spring 2018 in the 55-m array (11–24 April). Each tagged fish was measured to the nearest mm fork or total length (FL or TL) and then returned to the water as quickly as possible. Fish were released either at the surface or at depth using a descender device. The effect of descender device usage on survivorship and details on all tagged fish were reported in Bohaboy et al.^70^. The tagging protocol used in this study was approved by the Institutional Animal Care and Use Committee of the University of Florida and all methods were carried out in accordance with relevant guidelines and regulations. Three to 6 months after each tagging event, detection data were offloaded from retrieved acoustic receivers and sent to Vemco for Vemco Positioning System (VPS) geolocation estimation. Vemco provided position estimates as both latitude/longitude coordinates and relative northing (Y) and easting (X) in meters.

### Data analysis

#### Position error and filtering

Vemco-computed error sensitivity (precision; HPE, unitless) was provided for all VPS position estimates of tagged animals and transmitters as well as estimates of horizontal position errors (HPE_m_, m) for VR2Tx receivers which have measured GPS coordinates. Smith^84^ reported a statistical method to establish a relationship between HPE_m_ and HPE for VR2Tx receivers which can be applied to HPE estimates of tagged animals in order to estimate absolute position error (${HPE}_{m}^{*}$, m) of animal positions at the 95% confidence level. Calculation of ${HPE}_{m}^{*}$ as a function of HPE was performed for each of the 4 VPS datasets in this study separately, corresponding to each time receivers were retrieved (September 2016, March 2017, March 2018, and July 2018). We excluded all position estimates within the upper 5% of HPE values for each dataset before calculating ${HPE}_{m}^{*}$ to eliminate influence of extremely uncertain position estimates in our analyses^84^. Twice distance root mean squared (2DRMS) was calculated as twice the square root of the combined variance in X (VPS easting) and Y (VPS northing) for each 1-m increment of HPE averaged over all VRT2x receivers. The slope and intercept of the linear regression of HPE (the mid-point of each bin, i.e., each half meter) and 2DRMS were then applied to HPE estimates for transmitter tagged animals to produce an estimate of position error in m (${HPE}_{m}^{*}$).

For movement analyses such as daily reef use, reef switching, home range, and height above bottom, we filtered the data to exclude fish position estimates with error greater than 10 m (${HPE}_{m}^{*}$ > 10), which preserved 96.9% of animal position estimates among the 4 datasets. For the finer-scale analyses of fish distance to reef, we filtered the data to exclude fish position estimates with error greater than 5 m (${HPE}_{m}^{*}$> 5), which preserved 61.1% of animal position estimates (range 14.3 to 79.4% by dataset).

We validated VPS positional accuracy within several meters by performing a drift test in the 55-m array in July 2018 (detailed methods and results are included in supplementary materials). Positioning error can be introduced by the performance of the GPS equipment and service, as well as error in recording position of the deployed acoustic receivers. The boat used in this study is a working for-hire fishing vessel and all navigational equipment is routinely maintained and updated, so we are confident that horizontal GPS accuracy was within the U.S. Department of Defense 95% estimate of 3 m for “well designed GPS receivers”^85^. We minimized any additional error in recorded GPS positions by locating and recording the location of all acoustic receivers using the boat’s sonar depth sounder. Briefly, to validate array accuracy, an acoustic transmitter was suspended from the boat and moved slowly (approximately 0.25 m/s) through the array. Positions estimated by VPS were compared with recorded GPS positions to estimate absolute error. Average error was lowest in the center of the array (1.0 m) and higher at edges (3.7–7.9 m; Supplementary Table 5). Ideally, we would have been able to perform a drift test in each array or had stationary transmitters at measured positions (‘sentinel tags’) deployed through the duration of the study. Absent those observations, we believe our overall accuracy estimate of 4.1 m is conservative because the drift test was performed after the receivers had been deployed for several months allowing accumulation of biofouling which can reduce receiver efficiency^86^. We expect the positional accuracy of the 30-m array was greater because the spacing between receivers was less (450 m in the interior of the 30-m array vs. 500–600 m in the 55-m array) and the greater depths in the 55-m array added to the challenges inherent in deploying and pinpointing the locations of receivers in the open ocean.

#### Individuals included in the analyses

The ultimate fate and time of fate for each tagged fish was assigned by examining position and depth data over time following our previously published approach^70^. Possible fates for each tagged fish included predation (movements mirrored those of a large oceanic shark), emigration (the fish moved to the edge of the array and then exited), tag loss (the tag was stationary on the bottom within the array), surface mortality (failure to resubmerge following tag and release), and harvest by anglers or spear fishers (reported by fishers). A fish with 'unknown' fate was alive and present within the array up to a certain point in time, after which the disposition of the fish was ambiguous. For example, a transmitter tagged fish that apparently disappeared from the center of the array could be the result of transmitter malfunction, transmissions from a shed tag being blocked by reef structure, or unreported harvest by fishers. Tagged fish that were alive and present when receivers were retrieved in March 2017 and July 2018 were classified as 'at-large'. Only fish that were alive and present within the acoustic array for at least 4 days following release were included in behavioral analyses in order to exclude individuals that died as a result of capture and release. In addition, any positions that occurred after the assigned time of fate (e.g., long range movements following inferred predation) were excluded from the analyses.

#### Environmental covariates

Oceanographic conditions within and near the acoustic telemetry arrays were monitored throughout the study period. Hourly average significant wave height (m), sustained wind speed (m/s), peak wind speed (m/s), wind direction (degrees from north), and atmospheric pressure (mbar) were acquired from the National Data Buoy Center at Station 42012^87^, approximately 48 km west of the 30-m array, and Station 42040^88^, approximately 77 km west/southwest of the 55-m array. Average bottom temperatures (°C) were compiled from the data logs of the VR2Tx acoustic receiver located closest to the center of each array.

In addition to measured environmental conditions, the day of year (‘day’, ranging from 1 to 366) was included to explain seasonal effects. Similarly, the hour of the day (‘hour’) in UTC was included to describe the daily cycles in fish behavior or movement. Moon phase was calculated using the R package ‘lunar’^89^ and was expressed as radians × π^−1^ such that a value of 0 corresponds to the new moon, 0.5 to the first quarter, 1 to the full moon, and 1.5 to the last quarter. For illustration, we included dawn and dusk periods for each day calculated using the R package ‘suncalc’^90^. We define ‘dawn’ as the period between the commencement of morning nautical twilight and sunrise. We define ‘dusk’ as the period between sunset and the end of evening nautical twilight.

#### Reef use and height above bottom

For every fish position in the 10-m filtered dataset, we assigned the nearest reef as the one with the shortest Euclidean distance calculated in 2 dimensions. A fish was considered to have visited a reef if a position was within 20 m of the reef. We chose the 20 m cut-off as a conservative threshold to avoid falsely qualifying a fish position as visiting a reef when the fish may have been straying and returning to a neighboring reef. The average distance between nearest neighboring reefs in the 30-m and 55-m arrays was 350 and 520 m, respectively, and the minimum distance between neighboring reefs was 88 m in the 30-m array (reefs P and Q) and 210 m in the 55-m array (reefs a and b; Fig. 1B,C). We defined switching as a fish moving from one reef to another. Average time per reef was calculated for each fish as the number of days a fish was tracked within the array divided by the number of reef visits (N switches + 1).

Depth below the surface was given by the transmitter tag pressure sensor value and converted to estimated height above bottom for the filtered position estimates using sea floor depth from the NOAA National Centers of Environmental Information U.S. Coastal Relief Model^91^.

#### Reef fidelity

We used Kaplan–Meier nonparametric analysis^92^ to estimate the proportion of fish remaining on the original release reef (i.e., the proportion of fish surviving emigration to time *t*, $\widehat{{S}_{t}}$^56^). Fish observed moving to a different reef or exiting the acoustic telemetry array were treated as emigrants. Fish that were assigned fates of predation, tag loss, harvest, unknown, or were continuously present at the tagging reef up to the time when the study was terminated were right censored from the analysis. Site fidelity (*SF*) estimates are often given by mark-recapture studies as the proportion of total recaptured fish encountered at the release reef and by acoustic telemetry studies as the proportion of total monitored fish still present at the release site. The time interval over which site fidelity is calculated (*t*_*SF*_) is often specified as 1 year (thus annual site fidelity, *AF*) or is study-specific equal to the mean time at large for recaptured fish. Median residency time (*t* when $\widehat{{S}_{t}}$ = 0.5, henceforth *t*_*50%*_) is also reported for some studies of reef fish movement. Assuming simple exponential decay such that $\widehat{{S}_{t}}={N}_{t}/{N}_{0}={e}^{-t{R}_{E}}$ (Eq. 1), where *N*_*0*_ is the number of fish initially released on a given reef, *t* is in days, *N*_*t*_ is the number of fish remaining at the release reef at time *t*, and *R*_*E*_ is the daily emigration rate of fish away from the release reef, then $SF= {e}^{-{t}_{SF}{R}_{E}}$. Solving for R_E_, ${R}_{E}=-Ln(SF)/{t}_{SF}$, and substituting into Eq. (1) yields $\widehat{{S}_{t}}={e}^{tLn(SF)/{t}_{SF}}$. Hence, $SF={e}^{{t}_{SF}Ln(\widehat{{S}_{t}})/t}$, noting that *AF* is a special case where *t* = 365. We determined *t*_*50%*_ for the present study using Kaplan–Meier nonparametric survival analysis. We calculated *t*_*50%*_ from previous studies to enable comparison following $SF={e}^{{t}_{SF}Ln(0.5)/{t}_{50\%}}$, solving for *t*_*50%*_: ${t}_{50\%}= {t}_{SF}Ln(0.5)/Ln(SF)$.

#### Home range

We calculated movement-based kernel density estimates (KDEs) from Brownian Bridge modeled movements between VPS positions^93,94^ based on a bivariate-normal probability density kernel for 2 dimensions and extended to a trivariate-normal kernel for 3 dimensions^95^, as implemented in the R package 'MKDE'^96^. Movement-based KDEs are more appropriate for heavily auto-correlated geopositioning acoustic telemetry data than position-based methods that were developed with assumptions of independence of positions over time^97^. For movement-based KDEs, grid cells were 1 m^2^ or m^3^, with maximum delay between positions retained in the movement model of 1500 s and assumed X–Y and depth movement variances (${\sigma }_{xy}^{2}$ and ${\sigma }_{z}^{2}$ in Tracey et al.^96^) equal to 0.14 m^2^/s (the mid-point of observed red snapper swim speeds^70^). We defined home range as the 95% KDE of each fish (i.e., the central area or volume over which an animal has a 95% probability of occurring)^4,13,28,60^. Home ranges of fish were calculated over time intervals ranging from 1 h to several months (the duration of the study).

#### Factors affecting fish movement and behavior

We used generalized additive mixed-effects models (GAMMs) implemented in the R package 'mgcv'^98^ to examine the relationship between independent variables and fish home range, height above bottom, and reef association. GAMMs were fit separately for each species. Individual was treated as a random effect (via random intercept) and array (30-m vs. 55-m) was also included as a random intercept for red snapper (a random array effect was not necessary for gray triggerfish which were only tagged in the 30-m array). The error structure of the response variables was assumed based on the qualities of the data: natural log- transformed gamma distribution for hourly home range, normal distribution for height above bottom, and a lognormal distribution for distance to reef. Thin plate regression spline smoothing terms were applied to additive terms of wave height, wind speed (either sustained or peak), atmospheric pressure, bottom water temperature, and fish length. Cyclic cubic regression splines were specified for time of day (hour), day of year (day), moon phase, and wind direction. Two interaction terms were specified a priori and evaluated in the models: hour × day and moon phase × hour. The dimension of the basis (*k*) used to represent each smooth term was fixed at 10 for thin plate regression splines, 9 for cyclic cubic regression splines, and 17 for the interaction terms.

GAMMs were fit using maximum likelihood criteria and built using a forward (increasing number of variables) selection process. Each candidate GAMM was evaluated for parsimony according to the Akaike Information Criterion (AIC) based on the estimated practical degrees of freedom^98^ in each model. Variables were added to the GAMM models as long as each additional variable reduced AIC by at least 4. The number of input observations used to fit GAMMs of depth above bottom and distance to reef were limited by computer memory. We randomly selected 100,000 observations from the red snapper input datasets, 30,000 observations for gray triggerfish height above bottom, and 40,000 observations for gray triggerfish distance to reef. GAMMs for hourly home range were parameterized based on all available data (i.e., 63,000 for red snapper and 31,500 for gray triggerfish).

The effects of each independent variable in the selected model on expected and 95% CIs (± 1.96 × SE) of home range, height above bottom, and reef association were estimated with the predict.gam function in R package ‘mgcv’. For predictions of the variable of interest, remaining variables were held constant at the mean (wave height, wind speed, gust speed, atmospheric pressure, bottom water temperature, and fish length) or mode (day of year and wind direction) of observed values within the dataset. Hour and moon phase were fixed arbitrarily, usually at noon and first quarter, respectively, as noted.

### Ethics approval

The tagging protocol used in this study was approved by the Institutional Animal Care and Use Committee of the University of Florida and all methods were carried out in accordance with relevant guidelines and regulations.

## Supplementary Information

Supplementary Information.

## Data Availability

The datasets generated and analyzed during the current study are not publicly available because the precise locations of private artificial reefs within the study area are proprietary information. However, data are available from the corresponding author (erin.bohaboy@noaa.gov) on reasonable request.
