# Supplementary material for: Fine-scale movement of northern Gulf of Mexico red snapper and gray triggerfish estimated with three-dimensional acoustic telemetry
Source: Sci Rep. 2022 Aug 22;12:14274. doi: 10.1038/s41598-022-18451-x (PMC9395330; doi:10.1038/s41598-022-18451-x)
Supplement: Supplementary file 1 — Supplementary Information. [file 41598_2022_18451_MOESM1_ESM.pdf]

# Fine-scale Movement of Northern Gulf of Mexico Red Snapper and Gray Triggerfish Estimated with Three-Dimensional Acoustic Telemetry

Erin C. Bohaboy, Shannon L. Cass-Calay, and William F. Patterson III

## Supplementary Materials

Supplementary Data Tables and Figures

Supplementary Table 1. Acoustic tagged red snapper tracked during this study. Possible assigned fates are: emigrated from the array (em), mortality from predation (pred), unknown or ambiguous (unk), tag assumed to have fallen off of the fish (shed), and still alive and present within the array when the acoustic receivers were removed (at large).

| Fish ID | TL (mm) | N days w/ position estimates | N reefs visited | N switch | Average days per reef = N days/(N switch + 1) | Daily home range (95% KDE) | | | | | | Fate | Days between tagging and fate |
|---|---|---|---|---|---|---|---|---|---|---|---|---|---|
| | | | | | | $m^3$ (thousands) | | | $m^2$ (thousands) | | | | |
| | | | | | | med. | min. | max. | med. | min. | max. | | |
| 30-m array | | | | | | | | | | | | | |
| RS42 | 625 | 112 | 5 | 8 | 12.4 | 8.4 | 0.2 | 91.7 | 0.68 | 0.03 | 6.3 | em | 144.2 |
| RS53 | 393 | 28 | 1 | 0 | 28 | 8.6 | 3.2 | 57.1 | 0.78 | 0.47 | 4.1 | pred | 28.4 |
| RS16 | 466 | 38 | 1 | 0 | 38 | 8.9 | 3 | 31 | 0.7 | 0.4 | 2.1 | em | 38.4 |
| RS19 | 508 | 39 | 1 | 0 | 39 | 10.6 | 5.1 | 156.6 | 0.67 | 0.43 | 11.8 | unk | 39.1 |
| RS83 | 384 | 9 | 1 | 0 | 9 | 10.8 | 6 | 70.4 | 1.11 | 0.68 | 7.4 | pred | 8.8 |
| RS9 | 461 | 51 | 2 | 1 | 25.5 | 11.5 | 4.9 | 27.9 | 0.76 | 0.51 | 2.1 | em | 164.5 |
| RS62[1] | 410 | 16 | 1 | 0 | 16 | 12 | 5.9 | 20.8 | 0.9 | 0.54 | 1.5 | shed | 16.1 |
| RS78 | 387 | 11 | 5 | 9 | 1.1 | 12.2 | 4.8 | 65.9 | 1 | 0.61 | 7.2 | shed | 10.8 |
| RS6[1] | 376 | 48 | 1 | 0 | 48 | 14.9 | 8.1 | 60.1 | 1.1 | 0.68 | 4.4 | shed | 47.8 |
| RS7 | 406 | 42 | 1 | 0 | 42 | 15.5 | 6.4 | 178.1 | 1 | 0.59 | 13.3 | em | 77.4 |
| RS4 | 398 | 51 | 1 | 0 | 51 | 15.9 | 6.4 | 104.6 | 1.14 | 0.49 | 8.3 | em | 50.5 |
| RS63 | 395 | 38 | 1 | 0 | 38 | 17.2 | 3.7 | 92.4 | 1.54 | 0.5 | 5.1 | em | 41.1 |
| RS77 | 412 | 44 | 3 | 6 | 6.3 | 17.2 | 2.6 | 101 | 1.46 | 0.38 | 8.8 | em | 44.3 |
| RS72 | 372 | 17 | 3 | 3 | 4.3 | 17.4 | 1.5 | 43.9 | 1.43 | 0.19 | 3.5 | shed | 16.6 |
| RS82 | 394 | 23 | 2 | 1 | 11.5 | 18.7 | 5.6 | 43.6 | 1.45 | 0.48 | 2.9 | shed | 23.3 |
| RS45 | 335 | 5 | 2 | 1 | 2.5 | 20 | 12.5 | 44.2 | 1.53 | 1.34 | 3 | pred | 4.6 |
| RS52 | 366 | 4 | 1 | 0 | 4 | 20.5 | 8.6 | 135.6 | 1.42 | 0.75 | 11.9 | em | 4 |
| RS76 | 408 | 58 | 3 | 15 | 3.6 | 22.1 | 4.5 | 124.5 | 1.99 | 0.58 | 10.7 | em | 58.4 |
| RS57 | 331 | 190 | 2 | 2 | 63.3 | 25.2 | 2.4 | 302.4 | 2.19 | 0.41 | 27.4 | at large | 194.6 |
| RS60 | 370 | 27 | 1 | 0 | 27 | 26.4 | 5.7 | 59.8 | 2.02 | 0.73 | 4.4 | pred | 27.4 |
| RS46 | 366 | 36 | 1 | 0 | 36 | 28.6 | 7.1 | 132 | 2.15 | 0.6 | 8.2 | em | 35.5 |
| RS74 | 440 | 39 | 7 | 42 | 0.9 | 28.9 | 5.8 | 302.8 | 3.78 | 0.54 | 24.9 | em | 38.5 |

| Fish ID | TL (mm) | N days w/ position estimates | N reefs visited | N switch | Average days per reef = N days/(N switch + 1) | Daily home range (95% KDE) | | | | | | Fate | Days between tagging and fate |
|---|---|---|---|---|---|---|---|---|---|---|---|---|---|
| | | | | | | $m^3$ (thousands) | | | $m^2$ (thousands) | | | | |
| | | | | | | med. | min. | max. | med. | min. | max. | | |
| RS55 | 407 | 20 | 1 | 0 | 20 | 29 | 8.7 | 49 | 2.1 | 0.57 | 3.4 | shed | 19.5 |
| RS69 | 519 | 55 | 5 | 72 | 0.8 | 29.7 | 5.4 | 185.5 | 2.91 | 0.43 | 10.2 | shed | 55.2 |
| RS84 | 371 | 73 | 4 | 12 | 5.6 | 30.7 | 3.6 | 104.8 | 2.28 | 0.4 | 7.7 | shed | 73.7 |
| RS58 | 360 | 59 | 1 | 0 | 59 | 30.8 | 6.6 | 87.4 | 2.26 | 0.55 | 6.4 | shed | 59.1 |
| RS80 | 392 | 5 | 4 | 8 | 0.6 | 32 | 6.6 | 85.7 | 3.46 | 0.56 | 4.7 | em | 5.3 |
| RS59 | 372 | 189 | 2 | 144 | 1.3 | 32.8 | 7.7 | 256.1 | 2.45 | 0.6 | 18.1 | at large | 188.9 |
| RS75 | 427 | 5 | 4 | 3 | 1.3 | 33.6 | 7.2 | 93.7 | 2.52 | 0.68 | 6 | em | 4.5 |
| RS50 | 356 | 147 | 2 | 130 | 1.1 | 34.5 | 6.8 | 180.5 | 2.5 | 0.54 | 12.2 | pred | 147.1 |
| RS54 | 362 | 76 | 2 | 14 | 5.1 | 34.5 | 7.9 | 281.9 | 2.5 | 0.64 | 20.2 | shed | 76 |
| RS49 | 390 | 123 | 2 | 2 | 41 | 37.5 | 1.2 | 402.8 | 2.63 | 0.18 | 27.9 | shed | 123.1 |
| RS48 | 383 | 94 | 2 | 4 | 18.8 | 39.8 | 3.5 | 303.4 | 3.2 | 0.49 | 25.8 | em | 93.6 |
| RS65 | 395 | 95 | 1 | 0 | 95 | 46.1 | 6.2 | 192.1 | 3.28 | 0.45 | 15.1 | pred | 133.8 |
| RS64 | 395 | 92 | 5 | 19 | 4.6 | 50.8 | 12.5 | 482.9 | 3.31 | 0.82 | 35 | shed | 92.3 |
| 55-m array | | | | | | | | | | | | | |
| RS147 | 412 | 36 | 2 | 2 | 0.2 | 15 | 1.6 | 140.3 | 0.94 | 0.16 | 11 | shed | 36 |
| RS119 | 493 | 32 | 1 | 0 | 32 | 17.9 | 6.6 | 77.1 | 0.88 | 0.48 | 2.9 | shed | 31.5 |
| RS116 | 628 | 13 | 1 | 0 | 13 | 18.5 | 9.2 | 34.9 | 0.89 | 0.44 | 1.3 | shed | 13 |
| RS105 | 529 | 16 | 1 | 0 | 16 | 21.9 | 16.5 | 54.5 | 1.09 | 0.79 | 2.3 | shed | 15.5 |
| RS100[2] | 554 | 26 | 1 | 0 | 26 | 23 | 5.7 | 62.7 | 1.15 | 0.53 | 2.7 | shed | 25.5 |
| RS138 | 508 | 14 | 2 | 3 | 3.5 | 23.4 | 13.4 | 64 | 0.92 | 0.6 | 3.5 | em | 14.2 |
| RS128 | 479 | 68 | 1 | 0 | 68 | 25.2 | 1.4 | 132.6 | 1.28 | 0.11 | 3.9 | shed | 68.8 |
| RS129 | 439 | 88 | 1 | 0 | 88 | 26.2 | 9.6 | 71.5 | 1.41 | 0.56 | 3.3 | shed | 87.6 |
| RS148 | 594 | 19 | 1 | 0 | 19 | 27.1 | 18.2 | 171.6 | 1.31 | 0.83 | 11.8 | em | 19 |
| RS86 | 571 | 10 | 1 | 0 | 10 | 28.9 | 11 | 49.6 | 1.61 | 0.85 | 2.2 | shed | 9.7 |
| RS100B[2] | 595 | 29 | 1 | 0 | 29 | 30.4 | 16.6 | 139.1 | 1.47 | 0.94 | 8.5 | em | 28.5 |
| RS124 | 414 | 111 | 2 | 1 | 55.5 | 31.8 | 11 | 135.5 | 1.93 | 0.79 | 7.2 | at large | 110.9 |
| RS130 | 515 | 93 | 1 | 0 | 93 | 33.6 | 3.1 | 161 | 1.6 | 0.27 | 6.4 | em | 93.1 |
| RS125 | 569 | 40 | 1 | 0 | 40 | 34.7 | 9.7 | 119.4 | 1.89 | 0.67 | 4.7 | em | 39.9 |

| Fish ID | TL (mm) | N days w/ position estimates | N reefs visited | N switch | Average days per reef = N days/(N switch + 1) | Daily home range (95% KDE) | | | | | | Fate | Days between tagging and fate |
|---|---|---|---|---|---|---|---|---|---|---|---|---|---|
| | | | | | | $m^3$ (thousands) | | | $m^2$ (thousands) | | | | |
| | | | | | | med. | min. | max. | med. | min. | max. | | |
| RS120 | 615 | 70 | 2 | 1 | 35 | 35 | 13.8 | 79.8 | 1.61 | 0.67 | 3.8 | shed | 69.9 |
| RS136 | 761 | 83 | 5 | 37 | 2.2 | 35.5 | 1.5 | 612.8 | 1.91 | 0.14 | 38.3 | at large | 110.1 |
| RS163 | 443 | 79 | 1 | 0 | 79 | 36.1 | 12.6 | 221.9 | 1.89 | 0.82 | 14.2 | shed | 79.3 |
| RS91 | 627 | 36 | 1 | 0 | 36 | 36.8 | 13.9 | 76.5 | 1.82 | 0.76 | 4 | shed | 37 |
| RS155 | 623 | 64 | 2 | 1 | 32 | 37 | 7.9 | 135.8 | 1.6 | 0.43 | 5.8 | shed | 64.6 |
| RS135 | 502 | 8 | 1 | 0 | 8 | 39.9 | 19.6 | 79.9 | 2.27 | 1.05 | 3.6 | pred | 8.1 |
| RS158 | 464 | 98 | 2 | 1 | 49 | 40.3 | 5.8 | 86.8 | 1.77 | 0.38 | 3.9 | at large | 97.8 |
| RS131 | 472 | 103 | 2 | 2 | 34.3 | 41 | 3.1 | 371.2 | 1.84 | 0.3 | 25.8 | em | 103.1 |
| RS132 | 475 | 103 | 2 | 1 | 51.5 | 44.3 | 8 | 168.6 | 2.13 | 0.46 | 5.4 | shed | 104.7 |
| RS92 | 610 | 40 | 1 | 0 | 40 | 63.1 | 17.4 | 286.7 | 2.62 | 0.86 | 19.7 | shed | 40.2 |
| RS99 | 664 | 236 | 1 | 0 | 236 | 65.3 | 6 | 328.6 | 2.95 | 0.33 | 22 | at large | 323.6 |
| RS142 | 696 | 4 | 1 | 0 | 4 | 145.5 | 47.6 | 318.8 | 5.49 | 1.64 | 17.2 | em | 4.4 |

[1] RS6 was recaptured after having shed the acoustic transmitter and retagged as RS62.

[2] RS100 was recaptured after having shed the acoustic transmitter and retagged as RS100B.

Supplementary Table 2. Acoustic tagged gray triggerfish tracked during this study. Possible assigned fates are: harvested by fishermen (harv), emigrated from the array (em), mortality from predation (pred), unknown or ambiguous (unk), tag assumed to have fallen off of the fish (shed), and still alive and present within the array when the acoustic receivers were removed (at large).

| Fish ID | FL (mm) | N days w/ position estimates | N reefs visited | N switch | Average days per reef = N days/(N switch + 1) | Daily home range (95% KDE) | | | | | | Fate | Days between tagging and fate |
|---|---|---|---|---|---|---|---|---|---|---|---|---|---|
| | | | | | | $m^3$ (thousands) | | | $m^2$ (thousands) | | | | |
| | | | | | | med. | min. | max. | med. | min. | max. | | |
| GT37 | 431 | 16 | 1 | 0 | 16 | 4.7 | 2.6 | 8 | 0.4 | 0.29 | 0.5 | harv | 15.9 |
| GT41 | 330 | 100 | 2 | 2 | 33.3 | 5.5 | 2 | 17.3 | 0.51 | 0.25 | 1.1 | unk | 100.1 |
| GT23 | 348 | 141 | 3 | 67 | 2.1 | 7.2 | 2.2 | 42.3 | 0.55 | 0.21 | 3.6 | em | 141.2 |
| GT26 | 419 | 94 | 5 | 68 | 1.4 | 7.9 | 3.2 | 223.9 | 0.52 | 0.29 | 24.8 | em | 94.2 |
| GT21 | 485 | 4 | 1 | 0 | 4 | 11 | 6.8 | 83.1 | 0.64 | 0.55 | 5.2 | em | 4.3 |
| GT20 | 378 | 16 | 1 | 0 | 16 | 11.9 | 4.5 | 21.8 | 0.79 | 0.4 | 1.5 | harv | 16 |
| GT66 | 465 | 162 | 8 | 31 | 5.1 | 11.9 | 2.4 | 520.7 | 0.96 | 0.35 | 47.2 | at large | 194.9 |
| GT32 | 429 | 45 | 3 | 50 | 0.9 | 12.6 | 3 | 152.5 | 0.84 | 0.31 | 12.1 | pred | 44.5 |
| GT22 | 411 | 3 | 2 | 8 | 0.3 | 12.7 | 6.7 | 31.9 | 0.96 | 0.47 | 2.5 | em | 3.4 |
| GT29 | 471 | 266 | 18 | 625 | 0.4 | 35.8 | 2.1 | 757.1 | 2.62 | 0.23 | 56.3 | em | 266.6 |
| GT24 | 486 | 133 | 6 | 338 | 0.4 | 36.1 | 2.1 | 390.7 | 2.93 | 0.27 | 32.6 | em | 133.2 |
| GT39 | 420 | 39 | 2 | 46 | 0.8 | 42.1 | 1.8 | 652.6 | 3.1 | 0.23 | 53.2 | em | 39.1 |
| GT17 | 391 | 269 | 6 | 95 | 2.8 | 66.3 | 4.4 | 893.9 | 5.54 | 0.45 | 77.9 | em | 268.9 |
| GT34 | 481 | 328 | 11 | 592 | 0.6 | 100.1 | 1.3 | 1087.3 | 8.67 | 0.19 | 74.1 | shed | 333.3 |
| GT67 | 438 | 107 | 15 | 254 | 0.4 | 160.3 | 5.5 | 982.1 | 12.54 | 0.48 | 82.2 | em | 107.1 |

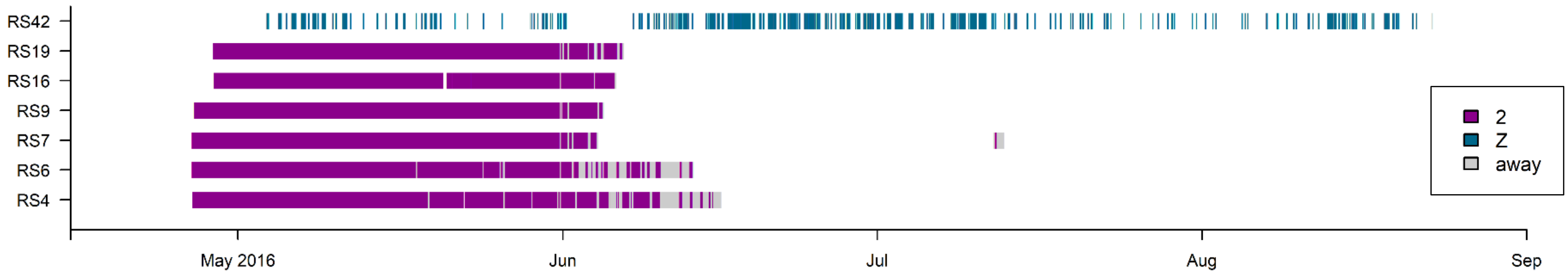


Supplementary Figure 1. Position history and daily closest reef for red snapper in the 30-m array from April 2016 through September 1, 2016. Days when the majority of fish positions were > 50 m from any reef are shown as "away".

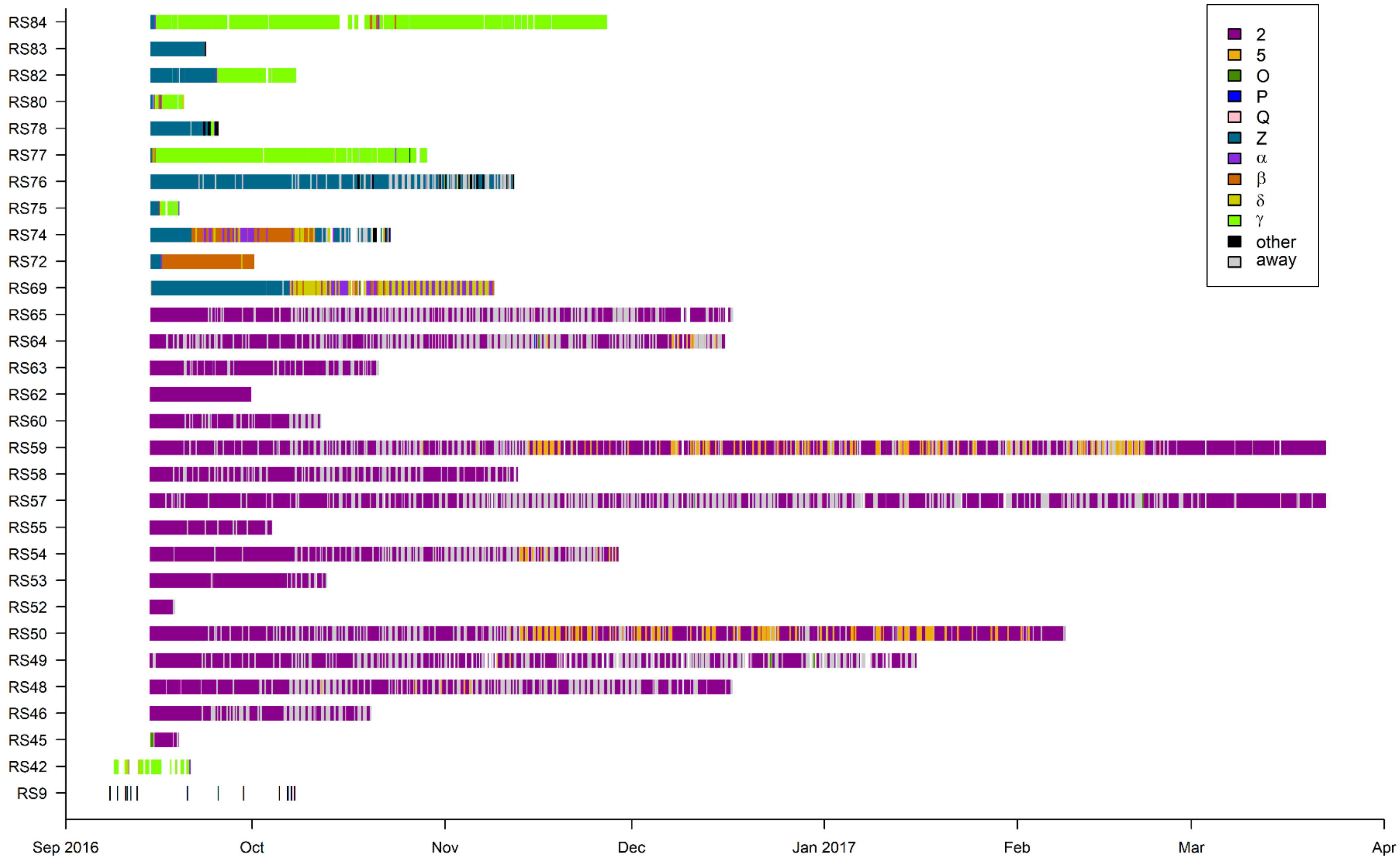


Supplementary Figure 2. Position history and resident reef for red snapper in the 30-m array from September 2016 through April 2017. Positions > 50 m from any reef are shown as "away". "Other" includes several reefs which combined accounted for << 1% of total positions (reefs 7, V, X, Y, and ε).

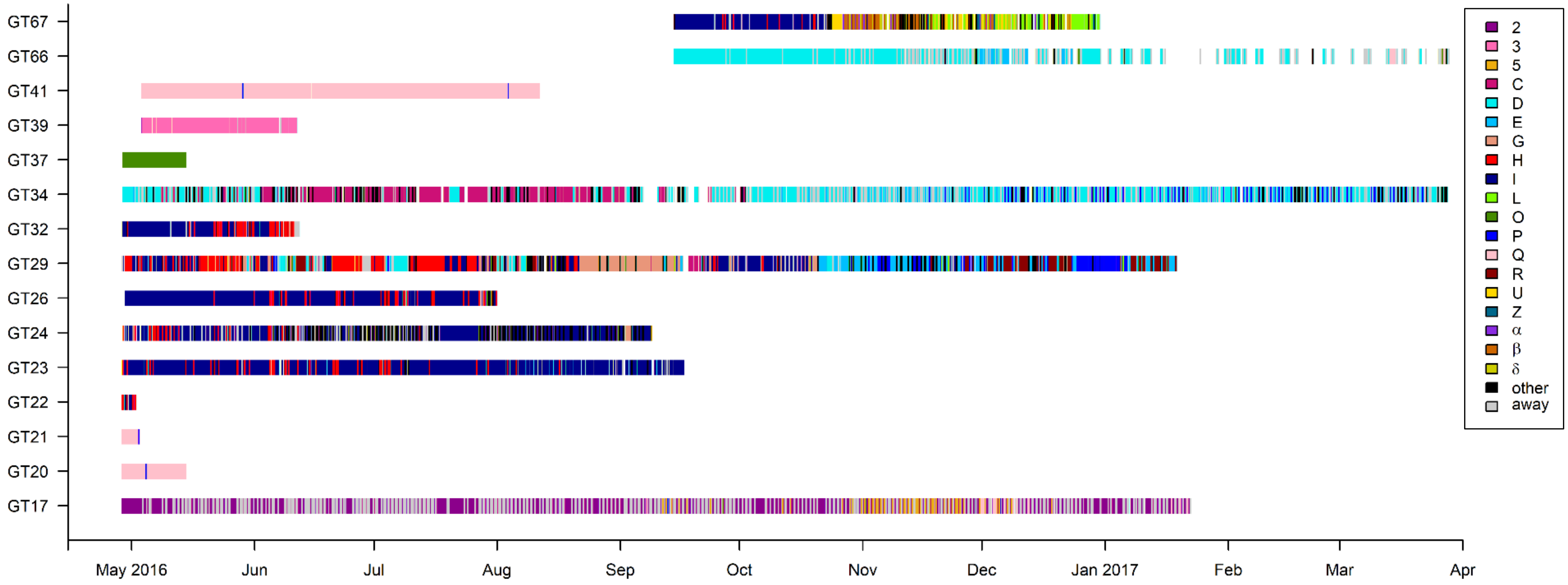


Supplementary Figure 3. Position history and resident reef for gray triggerfish in the 30-m array from April 2016 through April 2017. Positions > 50 m from any reef are shown as "away". "Other" includes several reefs which combined accounted for 1.5% of total positions (reefs 1, 6, A, B, F, J, K, M, S, X, Y, and γ).

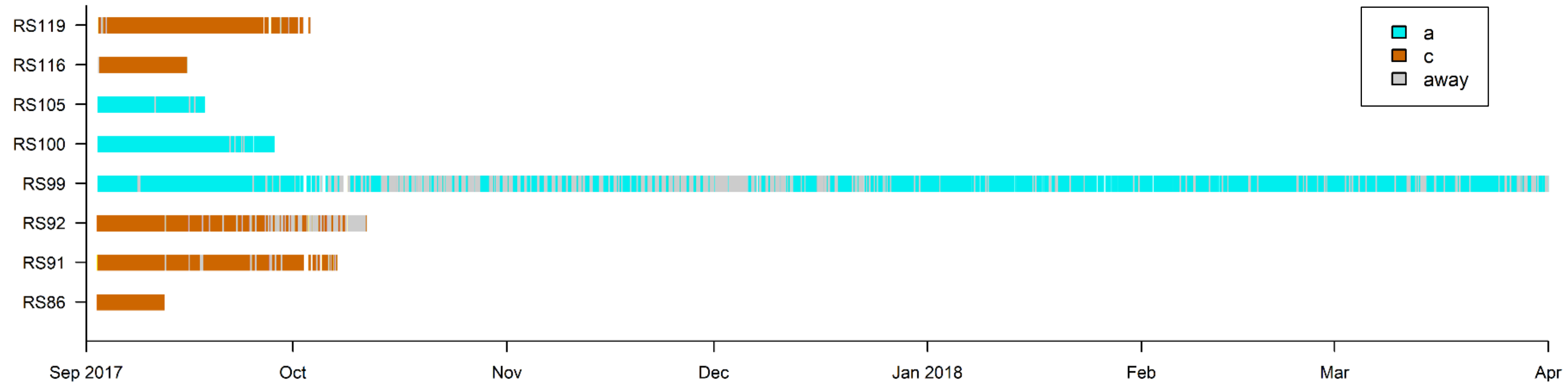


Supplementary Figure 4. Position history and resident reef for red snapper in the 55-m array from September 2017 through April 2018. Positions > 50 m from any reef are shown as "away".

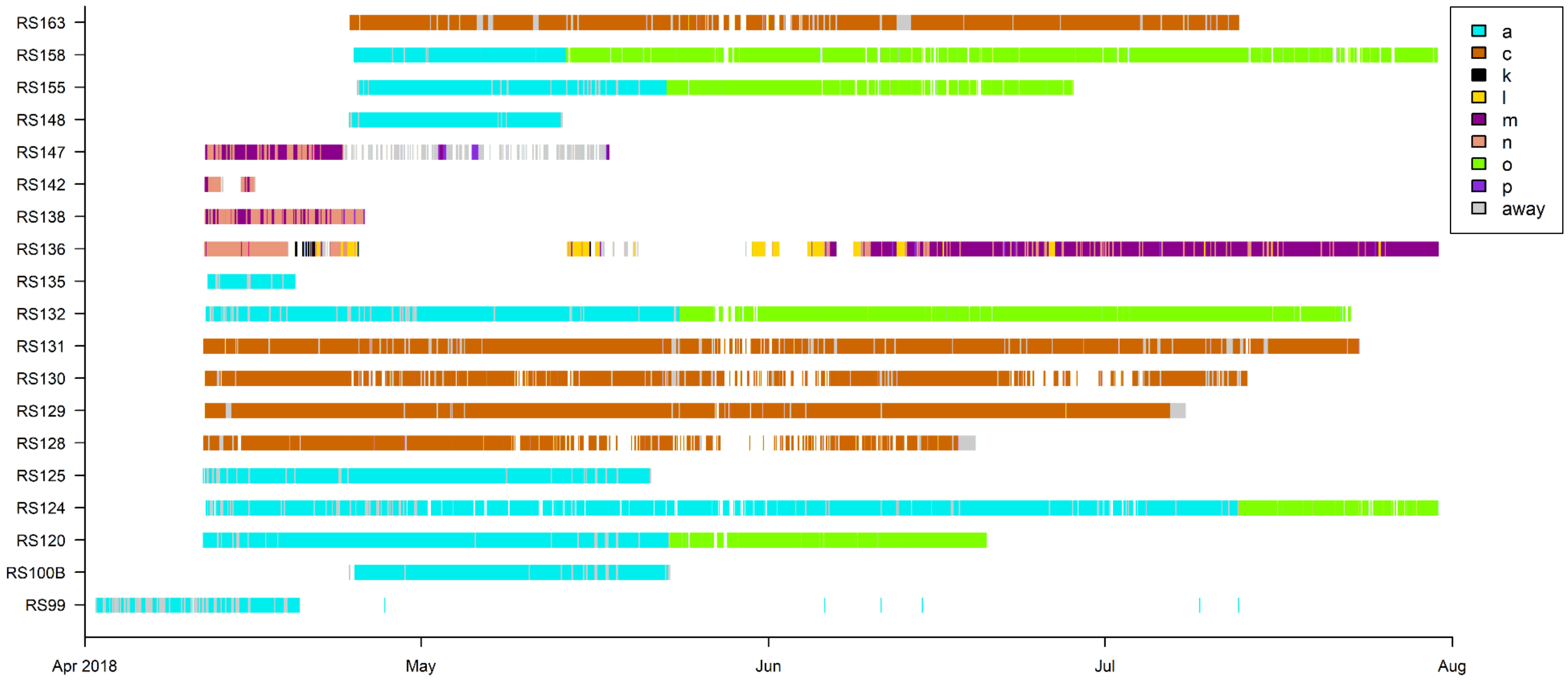


Supplementary Figure 5. Position history and resident reef for red snapper in the 55-m array from April 2018 through August 2018. Positions > 50 m from any reef are shown as "away".

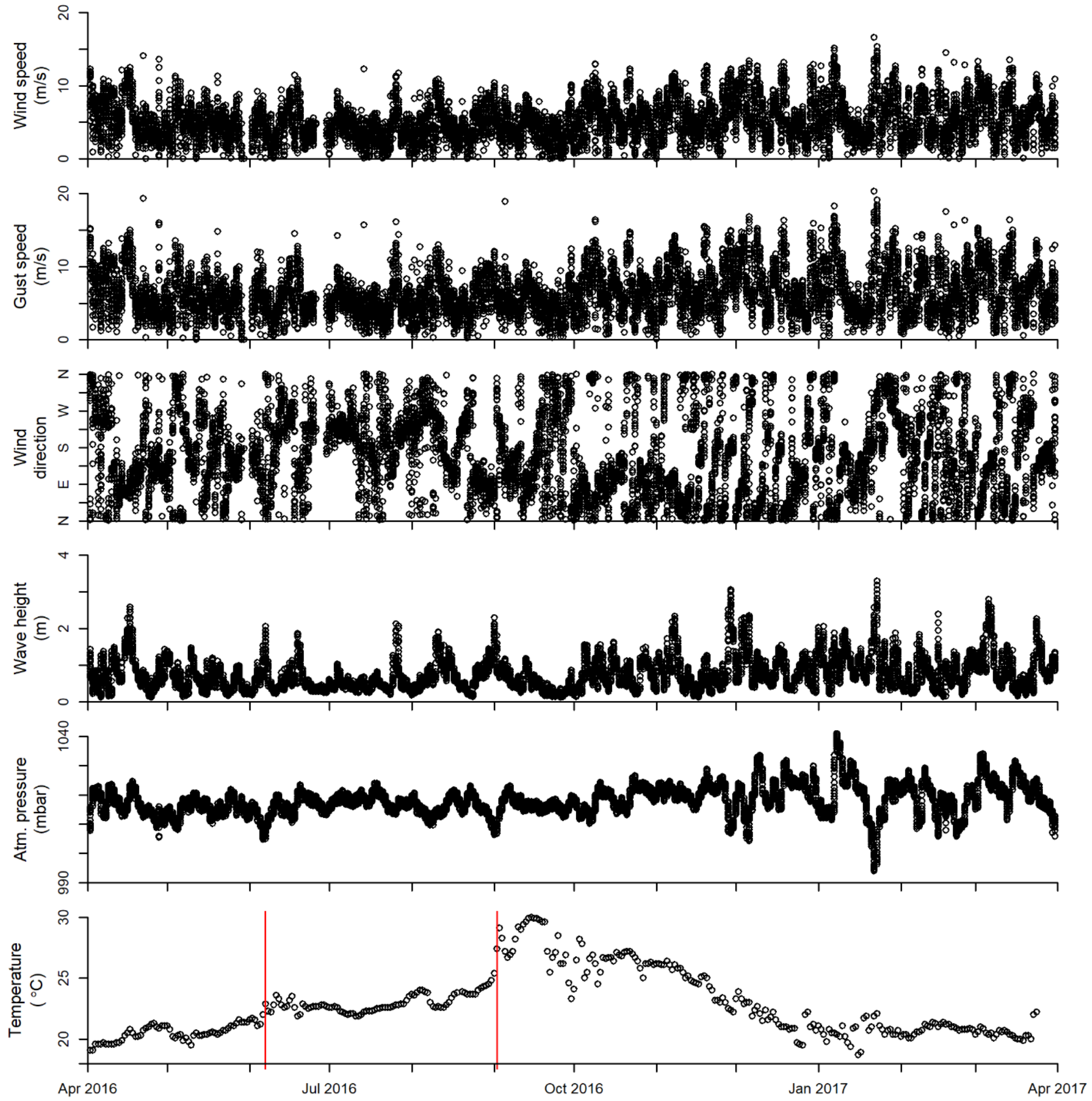


Supplementary Figure 6. Oceanographic conditions in the area of the 30-m array. Two named storms, Tropical Storm Colin and Hurricane Hermine, passed within 300 km of the array on 6 June 2016 and 2 September 2016 (vertical red lines on bottom pane). An unnamed storm system passed through the eastern GOM in late January 2017, causing high winds (>15 m/s), large waves (>3 m), and a drop in atmospheric pressure (below 994 mbar).

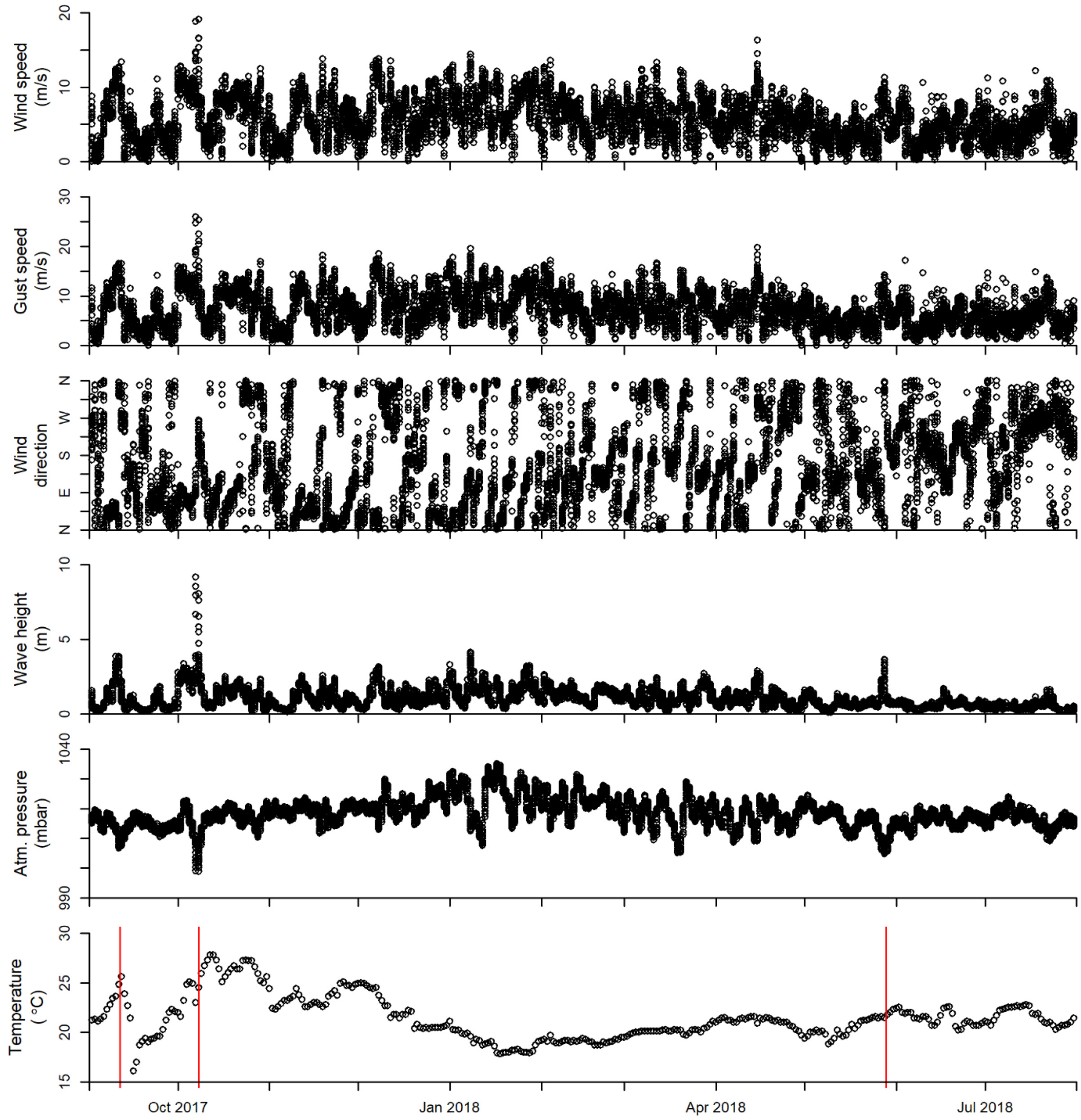


Supplementary Figure 7. Oceanographic conditions in the area of the 55-m array. Hurricane Irma, Hurricane Nate, and Tropical Storm Alberto affected conditions within the array on 11 September 2017, 8 October 2017, and 28 May 2018, respectively (vertical red lines on bottom pane).

Supplementary Table 3. Results of the GAMMs explaining variation of red snapper home range, height above bottom, and distance to reef.

| Response variable | Model component | Value | SE | *k* | Estimated degrees of freedom | Estimated p-value |
|---|---|---|---|---|---|---|
| **Home range ($m^2$)** | *Fixed effects* | | | | | |
| variance explained | intercept | 0.1611 | < 0.001 | 1 | 1 | < 0.001 |
| = 0.12 | temperature | 0.00853 | 0.002 | 1 | 1 | < 0.001 |
| | gust speed | 0.00156 | < 0.001 | 1 | 1 | 0.096 |
| | atm. pressure | 0.00095 | < 0.001 | 1 | 1 | 0.501 |
| | fish length | -0.00309 | < 0.001 | 1 | 1 | < 0.001 |
| | wave height | -0.000073 | < 0.001 | 1 | 1 | 0.921 |
| | *Random effects* | | | | | |
| | individual + array (intercept) | | | 61 | 61 | |
| | *Smooth terms* | | | | | |
| | day × hour | | | 15 | 11.75 | < 0.001 |
| | day | | | 8 | 7.61 | < 0.001 |
| | hour | | | 8 | 7.86 | < 0.001 |
| | temperature | | | 9 | 8.56 | < 0.001 |
| | gust speed | | | 9 | 4.50 | < 0.001 |
| | atm. pressure | | | 9 | 6.76 | < 0.001 |
| | length | | | 9 | 1 | < 0.001 |
| | wave height | | | 9 | 4.21 | < 0.001 |
| | moon phase × hour | | | 12 | 8.33 | < 0.001 |
| **Home range ($m^3$)** | *Fixed effects* | | | | | |
| variance explained | intercept | 0.12 | 0.0005 | 1 | 1 | < 0.001 |
| = 0.14 | temperature | 0.0037 | 0.001 | 1 | 1 | < 0.001 |
| | gust speed | 0.00073 | 0.0005 | 1 | 1 | 0.112 |
| | atm. pressure | 0.00069 | 0.001 | 1 | 1 | 0.521 |
| | fish length | -0.0044 | 0.0006 | 1 | 1 | < 0.001 |
| | *Random effects* | | | | | |
| | individual + array (intercept) | | | 61 | 61 | |
| | *Smooth terms* | | | | | |
| | day × hour | | | 15 | 11.81 | < 0.001 |
| | day | | | 8 | 7.7 | < 0.001 |
| | hour | | | 8 | 7.86 | < 0.001 |
| | temperature | | | 9 | 8.13 | < 0.001 |
| | gust speed | | | 9 | 4.08 | < 0.001 |
| | atm. pressure | | | 9 | 7.33 | < 0.001 |
| | length | | | 9 | 1 | < 0.001 |
| | moon phase × hour | | | 12 | 6.49 | < 0.001 |
| | moon phase | | | 8 | 6.45 | < 0.001 |
| **Height above** | *Fixed effects* | | | | | |
| **bottom** | intercept | 3.55 | 0.42 | 1 | 1 | < 0.001 |
| variance explained | temperature | 1.65 | 0.49 | 1 | 1 | < 0.001 |
| = 0.17 | wave height | -0.4 | 0.95 | 1 | 1 | 0.676 |
| | atm. pressure | 2.27 | 0.62 | 1 | 1 | < 0.001 |
| | wind speed | 0.68 | 0.41 | 1 | 1 | 0.096 |
| | *Random effects* | | | | | |
| | individual + array (intercept) | | | 73 | 73 | |
| | *Smooth terms* | | | | | |
| | day × hour | | | 15 | 11.95 | < 0.001 |
| | day | | | 8 | 7.99 | < 0.001 |
| | temperature | | | 9 | 8.94 | < 0.001 |
| | moon phase | | | 8 | 7.86 | < 0.001 |
| | wave height | | | 9 | 8.11 | < 0.001 |
| | atm. pressure | | | 9 | 8.49 | < 0.001 |
| | hour | | | 8 | 7.56 | < 0.001 |
| | wind direction | | | 8 | 7.44 | < 0.001 |
| | wind speed | | | 9 | 6.63 | < 0.001 |
| | moon phase × hour | | | 12 | 7.59 | < 0.001 |

Supplementary Table 3. *continued*. Results of the GAMMs explaining variation of red snapper home range, height above bottom, and distance to reef.

| Response variable | Model component | Value | SE | *k* | Estimated degrees of freedom | Estimated p-value |
|---|---|---|---|---|---|---|
| **Distance to reef** | *Fixed effects* | | | | | |
| variance explained | intercept | 2.74 | 0.04 | 1 | 1 | < 0.001 |
| = 0.17 | temperature | -0.23 | 0.13 | 1 | 1 | 0.070 |
| | wave height | -0.09 | 0.06 | 1 | 1 | 0.122 |
| | gust speed | -0.12 | 0.04 | 1 | 1 | 0.004 |
| | atm. pressure | -0.19 | 0.09 | 1 | 1 | 0.034 |
| | *Random effects* | | | | | |
| | individual + array (intercept) | | | 61 | 61 | |
| | *Smooth terms* | | | | | |
| | day × hour | | | 15 | 12.19 | < 0.001 |
| | day | | | 8 | 7.89 | < 0.001 |
| | hour | | | 8 | 7.72 | < 0.001 |
| | temperature | | | 9 | 8.08 | < 0.001 |
| | moon phase | | | 8 | 7.45 | < 0.001 |
| | moon phase × hour | | | 12 | 10.46 | < 0.001 |
| | wave height | | | 9 | 7.1 | < 0.001 |
| | gust speed | | | 9 | 5.49 | < 0.001 |
| | atm. pressure | | | 9 | 8.1 | < 0.001 |
| | wind direction | | | 8 | 5.6 | < 0.001 |

Supplementary Table 4. Results of the GAMMs explaining variation of gray triggerfish home range, height above bottom, and distance to reef.

| Response variable | Model component | Value | SE | *k* | Estimated degrees of freedom | Estimated p-value |
|---|---|---|---|---|---|---|
| **Home range ($m^2$)** | *Fixed effects* | | | | | |
| variance explained | intercept | 0.1650 | 0.002 | 1 | 1 | < 0.001 |
| = 0.26 | wave height | -0.0197 | 0.004 | 1 | 1 | < 0.001 |
| | temperature | 0.0050 | 0.003 | 1 | 1 | 0.103 |
| | atm. pressure | 0.0021 | 0.003 | 1 | 1 | 0.468 |
| | fish length | -0.0045 | 0.002 | 1 | 1 | 0.013 |
| | *Random effects* | | | | | |
| | individual (intercept) | | | 15 | 15 | |
| | *Smooth terms* | | | | | |
| | hour | | | 8 | 7.87 | < 0.001 |
| | day | | | 8 | 7.90 | < 0.001 |
| | day × hour | | | 15 | 11.02 | < 0.001 |
| | wave height | | | 9 | 7.73 | < 0.001 |
| | temperature | | | 9 | 7.95 | < 0.001 |
| | moon phase × hour | | | 12 | 6.73 | < 0.001 |
| | atm. pressure | | | 9 | 7.10 | < 0.001 |
| | fish length | | | 9 | 1.00 | 0.004 |
| | wind direction | | | 8 | 5.56 | < 0.001 |
| **Home range ($m^3$)** | *Fixed effects* | | | | | |
| variance explained | intercept | 0.12 | 0.001 | 1 | 1 | < 0.001 |
| = 0.28 | wave height | -0.02 | 0.003 | 1 | 1 | < 0.001 |
| | temperature | 0.0025 | 0.002 | 1 | 1 | 0.122 |
| | atm. pressure | 0.0009 | 0.002 | 1 | 1 | 0.622 |
| | gust speed | -0.0003 | 0.001 | 1 | 1 | 0.807 |
| | *Random effects* | | | | | |
| | individual (intercept) | | | 15 | 15 | |
| | *Smooth terms* | | | | | |
| | hour | | | 8 | 7.83 | < 0.001 |
| | day | | | 8 | 7.88 | < 0.001 |
| | day × hour | | | 15 | 14.72 | < 0.001 |
| | wave height | | | 9 | 8.27 | < 0.001 |
| | temperature | | | 9 | 7.33 | < 0.001 |
| | moon phase × hour | | | 12 | 6.95 | < 0.001 |
| | atm. pressure | | | 9 | 7.1 | < 0.001 |
| | wind direction | | | 8 | 5.51 | < 0.001 |
| | gust speed | | | 9 | 5.37 | < 0.001 |
| **Height above** | *Fixed effects* | | | | | |
| **bottom** | intercept | 2.60 | 0.46 | 1 | 1 | < 0.001 |
| variance explained | temperature | 3.59 | 0.53 | 1 | 1 | < 0.001 |
| = 0.17 | atm. pressure | -0.12 | 0.63 | 1 | 1 | 0.843 |
| | wind speed | 0.07 | 0.15 | 1 | 1 | 0.622 |
| | wave height | 0.18 | 0.17 | 1 | 1 | 0.269 |
| | *Random effects* | | | | | |
| | individual (intercept) | | | 15 | 15 | |
| | *Smooth terms* | | | | | |
| | day × hour | | | 15 | 12.38 | < 0.001 |
| | temperature | | | 9 | 8.78 | < 0.001 |
| | hour | | | 8 | 7.71 | < 0.001 |
| | moon phase | | | 8 | 7.24 | < 0.001 |
| | atm. pressure | | | 9 | 7.41 | < 0.001 |
| | moon phase × hour | | | 12 | 7.00 | < 0.001 |
| | wind direction | | | 8 | 5.71 | < 0.001 |
| | wind speed | | | 9 | 3.65 | < 0.001 |
| | day | | | 8 | 6.06 | < 0.001 |
| | wave height | | | 9 | 3.84 | < 0.001 |

Supplementary Table 4. *continued*. Results of the GAMMs explaining variation of gray triggerfish home range, height above bottom, and distance to reef.

| **Response variable** | **Model component** | **Value** | **SE** | ***k*** | **Estimated degrees of freedom** | **Estimated p-value** |
|---|---|---|---|---|---|---|
| **Distance to reef** | *Fixed effects* | | | | | |
| variance explained | intercept | 2.141 | 0.164 | 1 | 1 | < 0.001 |
| = 0.17 | temperature | -0.033 | 0.14 | 1 | 1 | 0.811 |
| | wave height | 1.462 | 0.246 | 1 | 1 | < 0.001 |
| | atm. pressure | -0.239 | 0.218 | 1 | 1 | 0.272 |
| | wind speed | 0.084 | 0.106 | 1 | 1 | 0.429 |
| | *Random effects* | | | | | |
| | individual (intercept) | | | 15 | 15 | |
| | *Smooth terms* | | | | | |
| | hour | | | 8 | 7.82 | < 0.001 |
| | temperature | | | 9 | 7.45 | < 0.001 |
| | day | | | 8 | 7.8 | < 0.001 |
| | day × hour | | | 15 | 14.65 | < 0.001 |
| | moon phase | | | 8 | 6.38 | < 0.001 |
| | wave height | | | 9 | 8.19 | < 0.001 |
| | atm. pressure | | | 9 | 7.45 | < 0.001 |
| | moon phase × hour | | | 12 | 8.54 | < 0.001 |
| | wind direction | | | 8 | 6.02 | < 0.001 |
| | wind speed | | | 9 | 6.29 | < 0.001 |

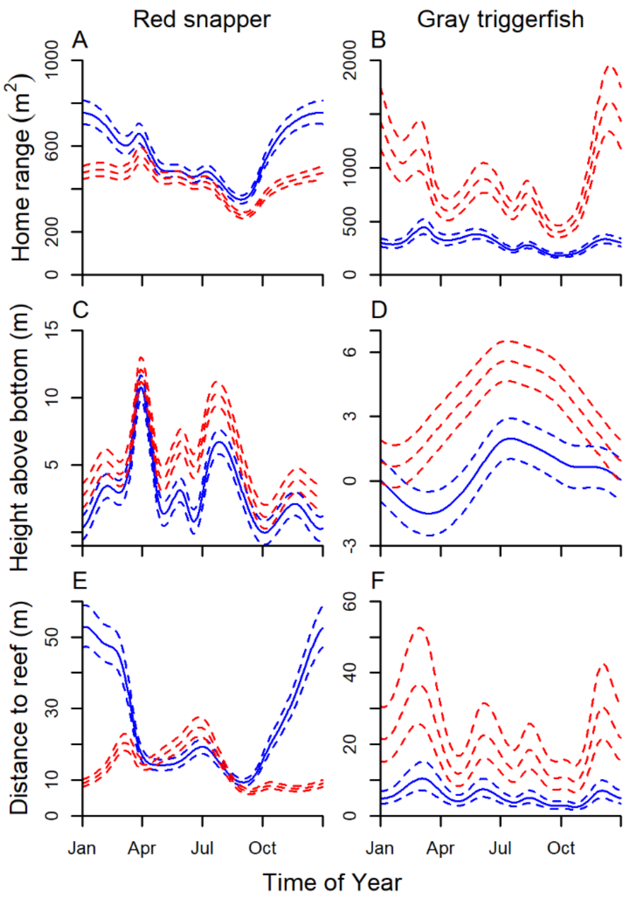


Supplementary Figure 8. GAMM predicted (solid line) and 95% CIs (broken line) of hourly home range (95% KDE; **A−B**), height above bottom (**C−D**), and distance to reef (**E−F**) of red snapper and gray triggerfish as a function of time of year. In each figure, time of day = 0000 is blue and 1200 is red.

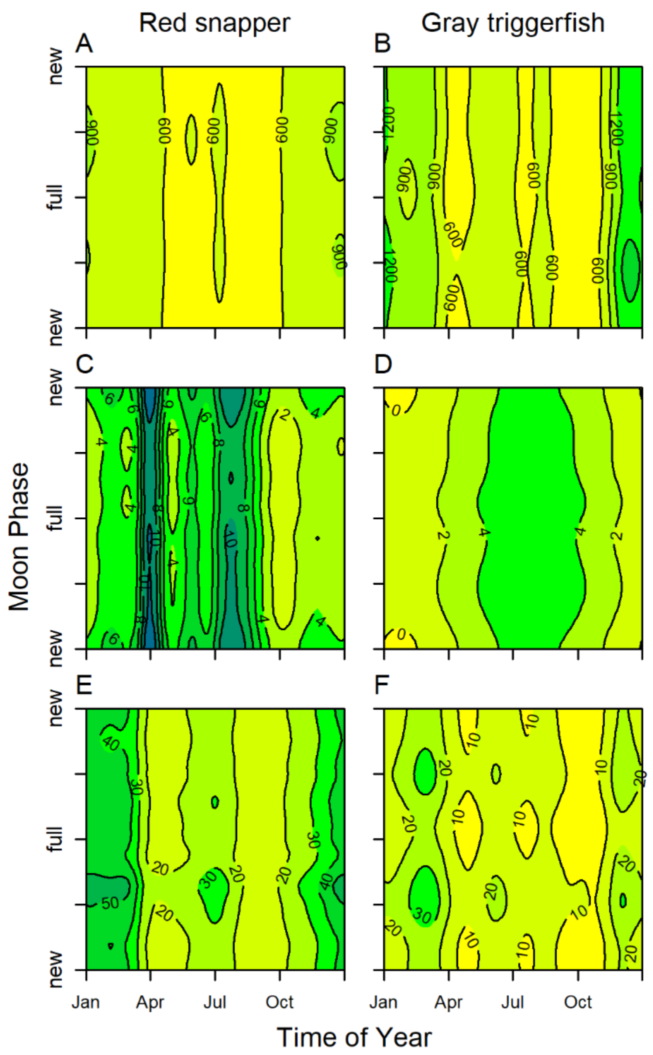


Supplementary Figure 9. GAMM predicted hourly home range (m$^2$; **A−B**), height above bottom (m, **C−D**), and distance to reef (m, **E−F**) of red snapper and gray triggerfish as a function of moon phase and time of year. Model predictions were calculated assuming 1800 and 1200 UTC-6 (dusk and noon) for red snapper and gray triggerfish, respectively.

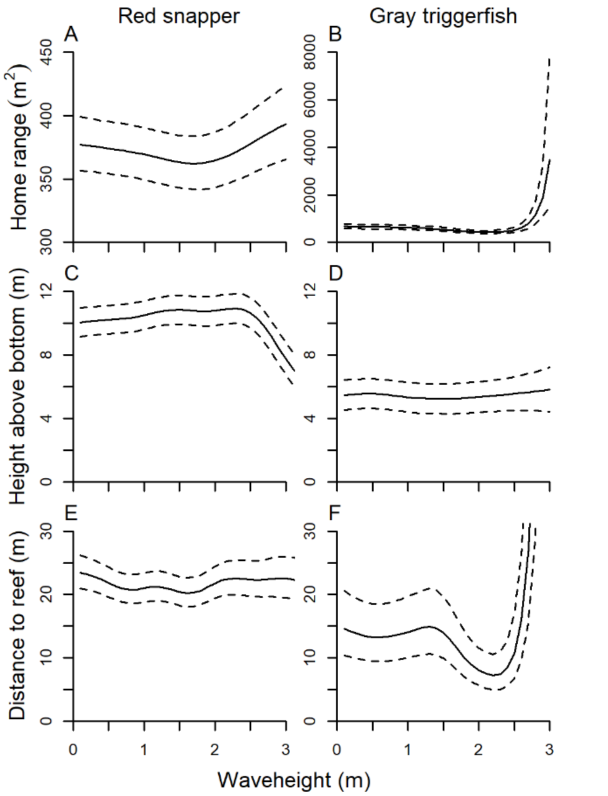


Supplementary Figure 10. GAMM predicted (solid line) and 95% CIs (broken line) of hourly home range (95% KDE; **A−B**), height above bottom (**C−D**), and distance to reef (**E−F**) of red snapper and gray triggerfish as a function of wave height.

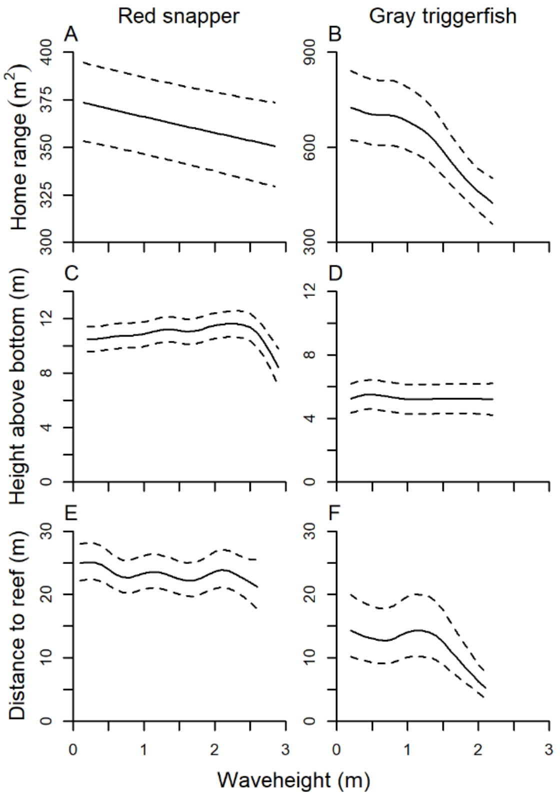


Supplementary Figure 11. GAMM predicted (solid line) and 95% CIs (broken line) of hourly home range (95% KDE; **A−B**), height above bottom (**C−D**), and distance to reef (**E−F**) of red snapper and gray triggerfish as a function of wave height. Position estimates outside the center 99% quantile for each variable were excluded from the model.

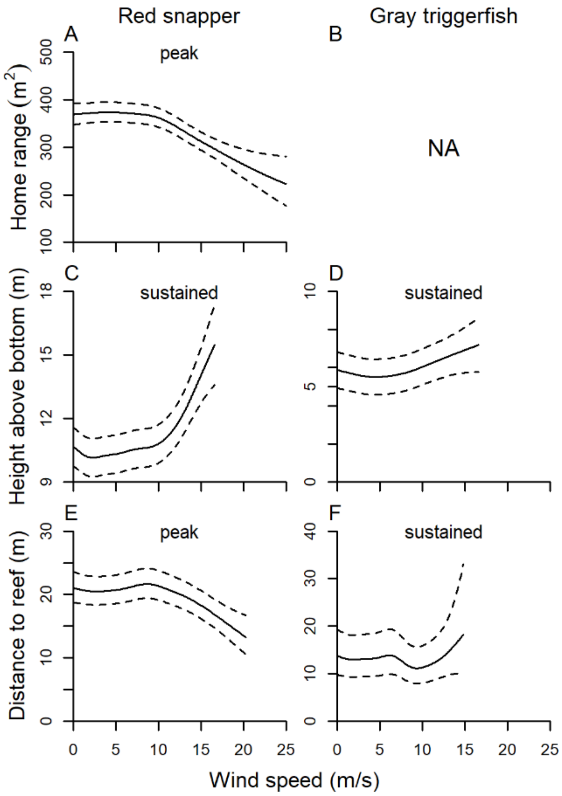


Supplementary Figure 12. GAMM predicted (solid line) and 95% CIs (broken line) of hourly home range (95% KDE; **A−B**), height above bottom (**C−D**), and distance to reef (**E−F**) of red snapper and gray triggerfish as a function of wind speed (either sustained or peak wind, as noted).

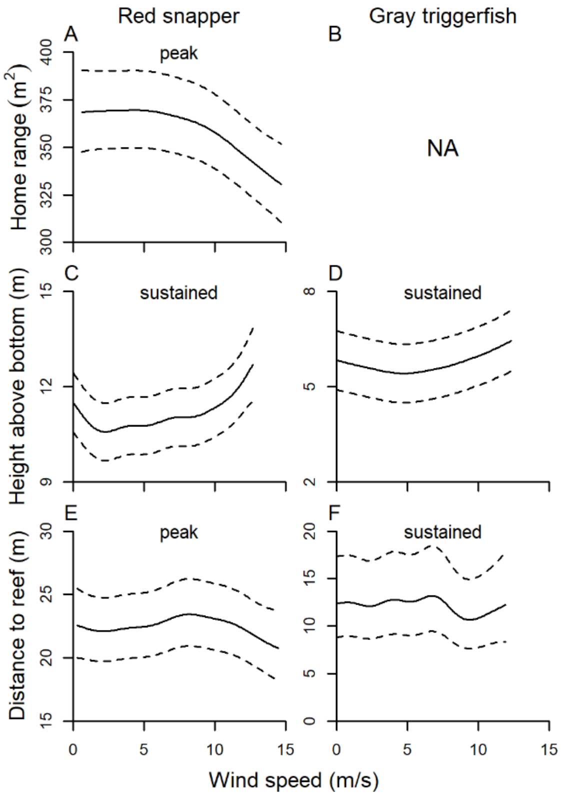


Supplementary Figure 13. GAMM predicted (solid line) and 95% CIs (broken line) of hourly home range (95% KDE; **A−B**), height above bottom (**C−D**), and distance to reef (**E−F**) of red snapper and gray triggerfish as a function of wind speed (either sustained or peak wind, as noted). Position estimates outside the center 99% quantile for each variable were excluded from the model.

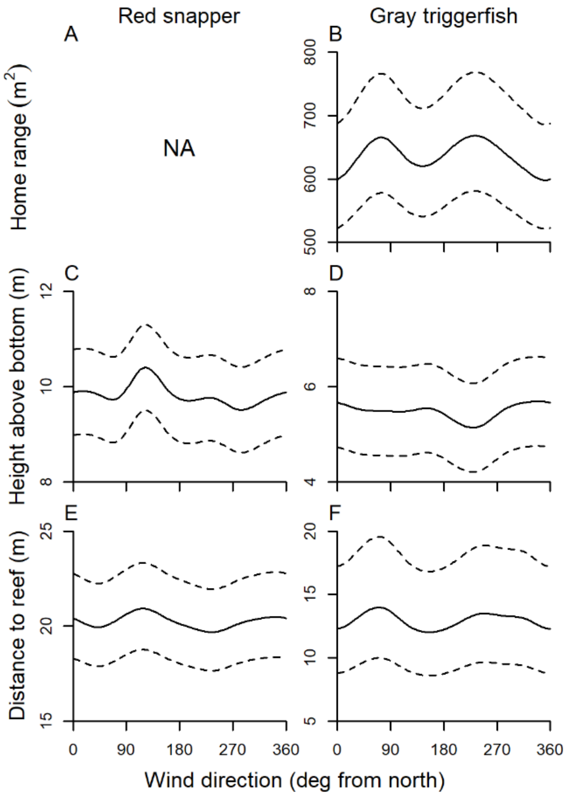


Supplementary Figure 14. GAMM predicted (solid line) and 95% CIs (broken line) of hourly home range (95% KDE; **A−B**), height above bottom (**C−D**), and distance to reef (**E−F**) of red snapper and gray triggerfish as a function of wind direction.

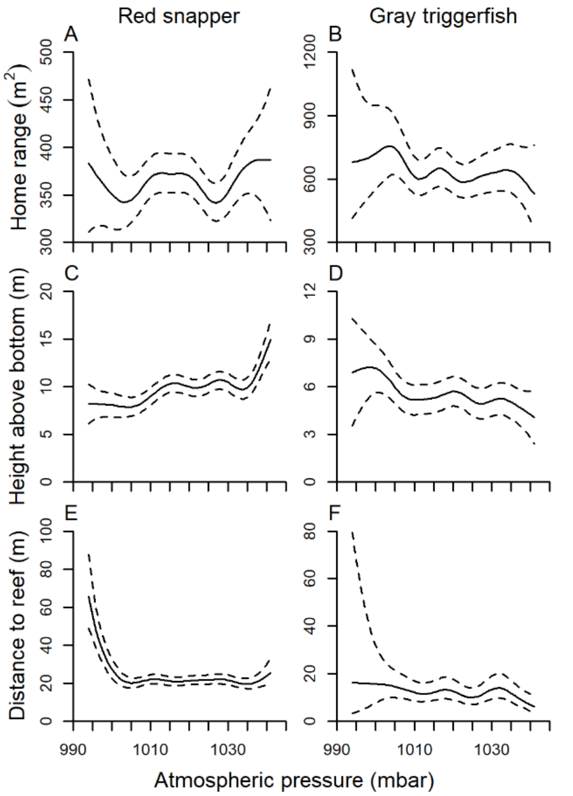


Supplementary Figure 15. GAMM predicted (solid line) and 95% CIs (broken line) of hourly home range (95% KDE; **A−B**), height above bottom (**C−D**), and distance to reef (**E−F**) of red snapper and gray triggerfish as a function of atmospheric pressure.

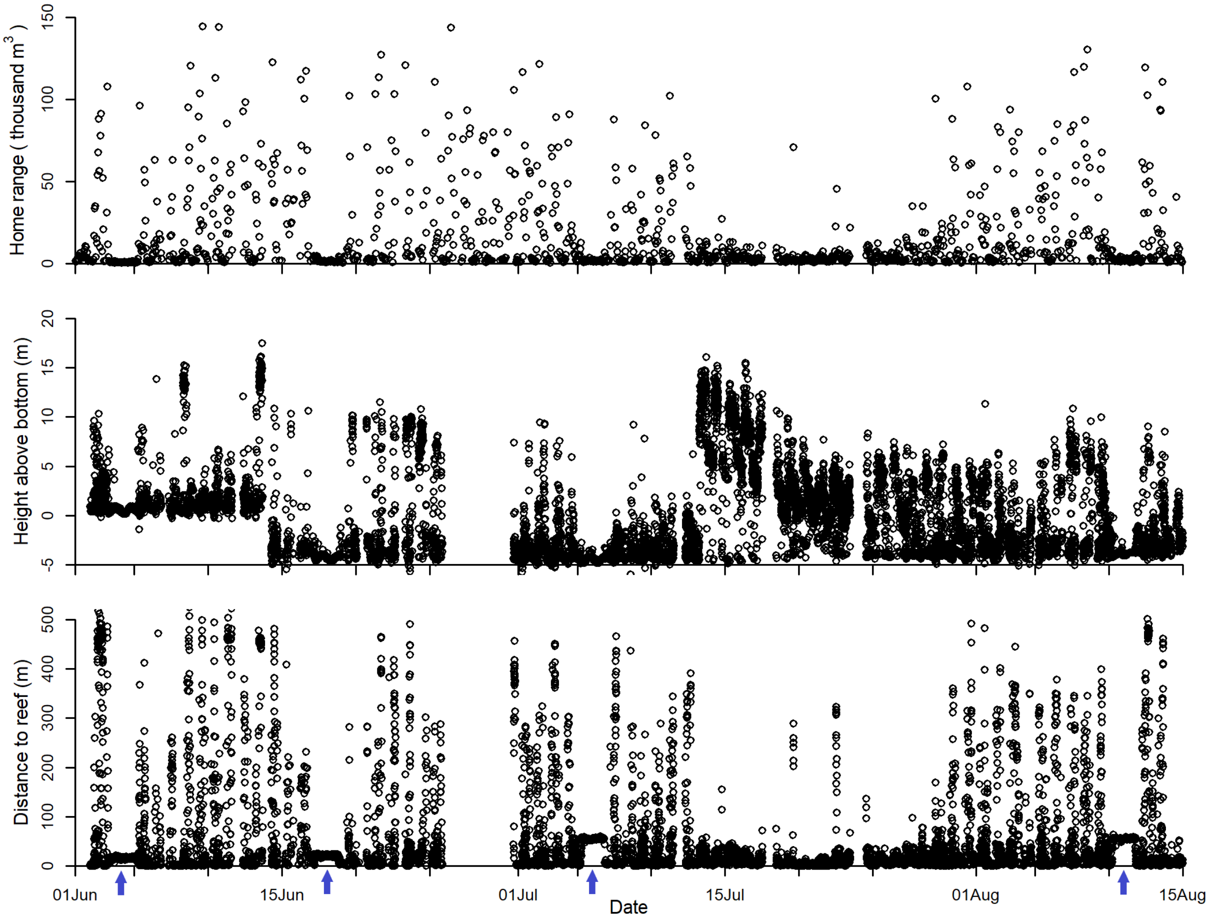


Supplementary Figure 16. Hourly home range (3-dimensional 95% KDE), height above bottom, and distance to reef of GT34 during the 2016 spawning season. Arrows note qualitative shifts in movement which persisted for approximately 2 days during each occurrence.

Array accuracy

*Methods*- We performed a "drift test" to validate positioning accuracy within the 55-m acoustic array by comparing VPS calculated coordinates to those recorded directly from the GPS positioning system on the boat. A Vemco V13T transmitter (identical to the transmitter used on tagged animals in this study, but with a shorter fixed delay between transmissions and a temperature rather than pressure sensor) was suspended approximately 3 m below the boat from the port side of the stern. The boat was allowed to drift for at least 10 minutes while the time, boat heading, and GPS coordinates from the ship's navigation system were recorded. The drift test was repeated at 5 different sites within the 55-m acoustic array: the center and each corner (NW, NE, SW, and SE; Fig. 17A). The GPS antenna is located on top of the wheelhouse in the center of the boat used for the drift test, which caused an offset between the recorded GPS location and the actual position of the transmitter. VPS transmitter positions were therefore corrected to the GPS antenna location based on the geometry of the boat (9.6 m and +18.4° relative to the bow) and the heading of the bow during each drift test (Fig. 17B).

*Results*-

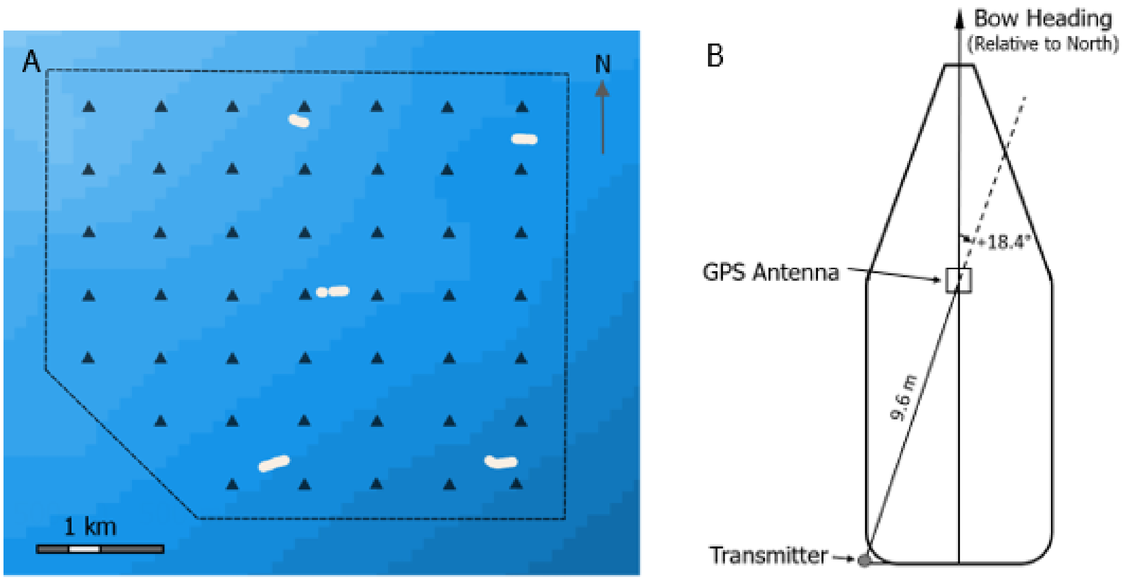


Supplementary Figure 17. Map and figure describing the drift test performed in the 55-m acoustic array to confirm the positioning accuracy of the array. A) Map showing the track of each drift test (white circles) within the array. The broken line indicates the approximate array extent and triangles indicate acoustic receiver positions. B) A diagram of the boat used for the

drift tests illustrating how transmitter locations were corrected to correspond to the GPS antenna according to the geometry of the boat and bow heading through the test.

Supplementary Table 5. Results of the drift test to evaluate positional accuracy of the 55-m array. Positioning efficiency is the percent of test tag transmissions that yielded VPS position estimates.

| Site | Start (UTC) | End (UTC) | n Positions | Positioning efficiency | Accuracy (m) | | HPE | |
|---|---|---|---|---|---|---|---|---|
| | | | | | mean | range | mean | range |
| NW | 15:21 | 15:33 | 7 | 10% | 7.9 | 4.1–10.4 | 3.9 | 3.8–4.0 |
| NE | 15:04 | 15:14 | 13 | 22% | 5.1 | 3.1–7.6 | 4.6 | 3.3–6.9 |
| Center | 13:57 | 14:11 | 9 | 11% | 1.0 | 0.0–3.7 | 3.9 | 3.0–5.4 |
| SW | 14:39 | 14:49 | 40 | 67% | 4.3 | 0.5–8.2 | 3.2 | 2.4–6.2 |
| SE | 14:20 | 14:30 | 31 | 52% | 3.7 | 0.0–6.4 | 3.3 | 2.6–4.4 |
| Overall | | | 100 | 30% | 4.1 | 0.0–10.5 | 3.5 | 2.4–6.9 |
